# Nanoscale frictional imaging of ferroelectric domains

**DOI:** 10.1126/sciadv.aee0247

**Published:** 2026-06-26

**Authors:** Seongwoo Cho, Hyeonbin Moon, Iaroslav Gaponenko, Eunnuri Cho, Céline Lichtensteiger, Ruben Skjelstad Dragland, Ji Hye Lee, Jan Schultheiß, Dennis Meier, Seunghwa Ryu, Seungbum Hong, Patrycja Paruch

**Affiliations:** ^1^ Department of Quantum Matter Physics, University of Geneva, 1211 Geneva, Switzerland.; ^2^ Department of Mechanical Engineering, Korea Advanced Institute of Science and Technology (KAIST), Daejeon 34141, Republic of Korea.; ^3^ G. W. Woodruff School of Mechanical Engineering, Georgia Institute of Technology, Atlanta, GA 30332, USA.; ^4^ Department of Materials Science and Engineering, Korea Advanced Institute of Science and Technology (KAIST), Daejeon 34141, Republic of Korea.; ^5^ Department of Materials Science and Engineering, Norwegian University of Science and Technology (NTNU), NO-7491 Trondheim, Norway.; ^6^ Advanced Institute of Convergence Technology, Seoul National University, Suwon 16229, Republic of Korea.; ^7^ Faculty of Physics and Center for Nanointegration Duisburg-Essen (CENIDE), University of Duisburg-Essen, Duisburg, Germany.; ^8^ Research Center Future Energy Materials and Systems, Research Alliance Ruhr, 44780 Bochum, Germany.; ^9^ Department of AX, Korea Advanced Institute of Science and Technology (KAIST), Daejeon 34141, Republic of Korea.; ^10^ KAIST InnoCORE PRISM-AI Center, Korea Advanced Institute of Science and Technology (KAIST), Daejeon 34141, Republic of Korea.

## Abstract

Nanoscale control of electromechanical coupling transforms frictional information into a direct probe of ferroelectric polarization. Here, we show that an asymmetric friction response induced by high contact forces applied with a scanning probe microscopy tip enables spatially resolved visualization of ferroelectric polarization in thin films and single crystals. In agreement with multifield coupled continuum mechanical simulations, our results reveal that this friction asymmetry results from flexoelectrically induced polarization either competing with or enhancing the ferroelectric polarization, thus giving mechanical information on its orientation. This technique, which we term polarization-derived friction microscopy, allows rapid, low-cost, and voltage-free imaging of ferroelectric domains, thereby minimizing electrostatic measurement artifacts and achieving high scan rates beyond 14 frames per second. Our work opens broad opportunities for high-throughput functional imaging of surfaces with extensive potential applications such as voltage-free data acquisition and studies of polarization dynamics in complex environments.

## Teaser

Friction asymmetry induced by contact force allows rapid, voltage-free imaging of ferroelectric polarization.

## INTRODUCTION

Electromechanical coupling—the interconversion between electric polarization and mechanical deformation—is of great importance for the fundamental understanding of functional ferroics and enabling their applications. This coupling can manifest in several forms: piezoelectricity (*1*, *2*), where mechanical stress induces polarization; flexoelectricity (*3*–*5*), which involves polarization due to strain gradients that break centrosymmetry; and electrostriction (*6*), characterized by material deformation in response to an applied electric field. Since electrostriction lacks a corresponding inverse effect, mechanical deformation of electromechanically coupled materials typically results in electrical responses attributable to either piezoelectricity or flexoelectricity, depending on whether the stress is homogeneous or inhomogeneous. Controlling these electromechanical properties, along with their combinations, substantially enhances the functional potential of materials, revealing previously unexplored capabilities (*7*, *8*). For instance, the recently observed polarization-sensitive wear behavior of ferroelectric surfaces (*8*) results from an intricate interaction between piezoelectric and flexoelectric effects, observable only under optimized strain gradient conditions. In addition, the flexoelectric effect itself can enable mechanical control of ferroelectric domains, with ferroelectric polarization and domain patterns reversibly switched through flexoelectric imprinting induced by either the tip or the substrate, thereby eliminating the need for any applied electric field (*9*–*11*).

Advancements in atomic force microscopy (AFM) have been instrumental in exploring electromechanical coupling at the nanoscale, providing unparalleled insights into the surface structure and local physical properties of materials (*7*–*9*, *12*–*15*). This coupling is particularly prominent in ferroelectric systems, where distinct polarization states coexist, and the ability to manipulate local polarization through external electric fields or strain gradients is essential for their functional behavior. Piezoresponse force microscopy (PFM) (*16*, *17*) has become a critical tool to visualize such domains, using the converse piezoelectric effect. In this technique, a subcoercive ac electrical bias applied to a conductive probe induces local oscillating surface deformation, whose amplitude and phase (relative to the excitation signal) are measured using lock-in amplification and serve as imaging markers. Although PFM is a well-known technique for studying ferroelectric materials (*18*, *19*), it can sometimes suffer from electrically induced measurement artifacts (*17*, *20*), which may lead to inaccurate interpretation of the data.

Here, we demonstrate that comparable domain visualization can be achieved directly by using the differences in frictional response, without the need to probe electrically induced strain. Oppositely polarized domains present different surface physics and chemistry (*21*, *22*), which can result in variations in frictional properties. In addition, the highly inhomogeneous stress applied by the AFM tip induces substantial local electromechanical responses, further altering the surface frictional properties based on polarization orientation. As a result, the frictional characteristics of the ferroelectric material can be effectively used for domain visualization, offering an alternative to relying solely on the converse piezoresponse. By applying sufficiently high stress to accentuate flexoelectric responses and effectively remove surface adsorbates, our approach harnesses these frictional differences as a robust, intrinsic indicator of ferroelectric polarization orientation.

First, we demonstrate that simple frictional imaging using lateral force microscopy (LFM) (*23*)—at sufficiently high loading forces to minimize contributions from surface chemistry effects—allows direct frictional visualization of ferroelectric domains. We successfully apply this approach, which we term polarization-derived friction microscopy (PdFM), across several representative ferroelectric materials, including PbTiO_3_, LiNbO_3_, BiFeO_3_, and ErMnO_3_, thereby establishing its universality. Here, by exploring the LFM response across a wide range of key parameters including stress, electrostatic boundary conditions, relative humidity, and scan rate, we show that under high strain gradients, the asymmetric friction of ferroelectric domains originates primarily from flexoelectrically induced mechanical asymmetry and does not involve surface water (which can, however, play a key role at lower strain gradients). This insight not only clarifies the mechanisms governing frictional behavior in ferroelectric materials but also underscores the pivotal role of flexoelectrically induced polarization for imaging, as well as manipulating ferroelectric domains. Moreover, PdFM, being a voltage-free imaging technique that requires no signal amplification, conductive coating, or electrical wiring, enables rapid and low-cost visualization of polar states while effectively suppressing electrostatically driven artifacts.

## RESULTS

### Concept and modeling of PdFM

**Fig. 1.**
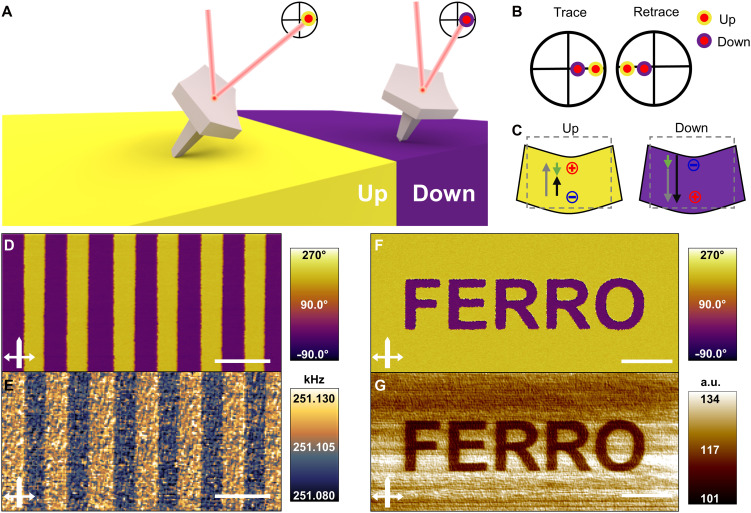
Nanoscale frictional imaging of ferroelectric domains: PdFM. (**A** and **B**) Schematic overview of frictional imaging of ferroelectric domains. The frictional response under high strain gradients, locally induced by the AFM tip, varies depending on the polarization configuration of the ferroelectric surface. The friction signal, monitored via a PSPD, changes on the basis of the polarization orientation of the domains during trace and retrace contact scanning. On the PSPD display, yellow and purple outlines around the red laser dot indicate signals from up and down domains, respectively. (**C**) Schematic illustration of strain gradient–induced flexoelectric polarization (green arrows) modifying the initial ferroelectric polarization (gray arrows), resulting in an asymmetric net polarization state (black arrows). (**D** and **E**) Vertical PFM phase (D) and contact resonance frequency imaging (E) confirm the mechanical asymmetry of up and down domains. (**F** and **G**) Frictional visualization of ferroelectric domains. Vertical PFM phase (F) and friction image (G) of artificially patterned up and down domains. During the friction imaging process, a 5-μN loading force was applied to the AFM tip with no electrical bias, demonstrating that in PdFM, domain structures can be discerned under purely mechanical stimulus. Scale bars, 1 μm [(D) and (E)] and 2 μm [(F) and (G)]. All measurements taken on PbTiO_3_ thin film. All images were acquired with the fast scan direction (white arrows) oriented perpendicular to the cantilever axis. a.u., arbitrary units.

Figure 1 illustrates the concept of frictional imaging to identify ferroelectric domains using PdFM. As schematically illustrated in Fig. 1 (A and B), cantilever tilt during contact imaging at high loading forces is more pronounced in ferroelectric up domains than in down domains. This torsional motion of the cantilever, reflecting the local frictional properties of the sample surface, is monitored via the lateral signal from the position-sensitive photodiode (PSPD) and is higher in up domains in PdFM (Fig. 1B). This asymmetric frictional response is due to the flexoelectric effect (Fig. 1C)—a universal phenomenon in all dielectrics and occasionally in metals (*3*, *24*–*27*), but particularly prominent in ferroelectrics, which have the highest values of flexoelectric coupling coefficients (*4*, *5*, *28*). Given the large strain gradients established by the local and highly inhomogeneous application of stress, the flexoelectric polarization becomes markedly enhanced and comparable to the piezoelectric polarization. Crucially, with respect to the surface normal of the (001) orientation, this flexoelectrically induced polarization is always down-oriented under the nanoscale AFM tip, while the piezoelectric response is intrinsically equivalent to the ferroelectric polarization, simply changing its orientation in down versus up domains. Therefore, the two codominant electromechanical effects either enhance or counteract each other, leading to different effective surface polarization (see fig. S1) and, thus, different mechanical and frictional properties. These differences can serve as imaging markers for ferroelectric polarization, allowing direct visualization of up versus down domains.

To explore our idea of visualizing ferroelectric domains through flexoelectrically induced frictional contrast, we start from ferroelectric PbTiO_3_ thin films [80-nm PbTiO_3_ on SRO/STO (001)], which have a uniform out-of-plane up (monodomain) orientation after growth. The surface AFM topography, vertical PFM amplitude, and phase of the PbTiO_3_ thin film after electrical switching of target domain patterns can be compared in fig. S2, while Fig. 1D shows PFM phase imaging after patterning of alternating ferroelectric up and down stripe domains by applying electrical bias to the AFM tip. As can be seen in the corresponding contact resonance frequency image (Fig. 1E), we observe a mechanical asymmetry between the up and down domains, with the latter showing a stiffer response (higher contact resonance frequency), consistent with findings from previous studies (*7*, *29*). When the word “FERRO” is written by contact scanning with a positively biased tip according to a predetermined down-domain pattern (PFM phase, Fig. 1F), the resulting friction image (Fig. 1G), acquired under a force of 5 μN at a scan speed of 146.48 μm/s, reproduces the same features with comparable resolution, confirming the ability of PdFM to visualize and map ferroelectric domains.

We note that no electrical bias is applied to the AFM tip during the PdFM scan, making it a voltage-free method, which can substantially reduce electrically induced measurement artifacts. Because PdFM does not rely on electrical current flow, barrier effects such as Schottky barriers—which often dominate the electrical response in conventional measurements—do not play a role in this approach. PdFM also simplifies the experimental setup by eliminating the need for conductive coating of the AFM probe, thereby minimizing potential artifacts from coating materials. In addition, PdFM operates entirely without electrical wiring or a back electrode, allowing straightforward implementation even on samples with complex geometries. This wiring-free nature also makes the technique well suited for studies under extreme environmental conditions—such as cryogenic or high-temperature measurements—where electrical connections are difficult to maintain. Furthermore, it eliminates the need for any signal amplifiers, such as lock-in amplifiers used for functional imaging of ferroelectrics including PFM (*17*), Kelvin probe imaging (*30*), and noncontact heterodyne electrostrain force microscopy (*31*). A current amplifier, as used for charge gradient microscopy (*32*), is likewise not required.

**Fig. 2.**
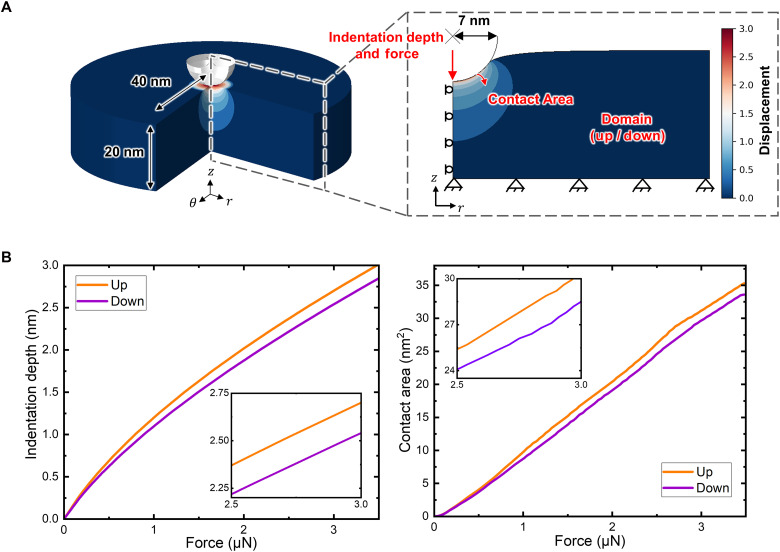
Axisymmetric finite element simulation of polarization-dependent contact mechanics. (**A**) Geometry and boundary conditions of the axisymmetric model with a rigid spherical indenter. (**B**) Representative depth-force (left) and contact area-force (right) curves for up- and down-poled domains. Results shown here correspond to $f_{1}=f_{2}=10\;C/\text{nm}$.

To clarify the nanoscale origin of the polarity-dependent friction asymmetry observed in PdFM, we performed axisymmetric finite-element simulations incorporating elasticity, piezoelectricity, and flexoelectricity. The computational setup consisted of a rigid spherical indenter pressing into a ferroelectric half-space under hard normal and frictionless tangential contact conditions (Fig. 2A). The model dimensions (40 nm by 20 nm) and indentation depth (≤3 nm) were chosen to suppress boundary effects and remain within the small-strain regime. Up- and down-poled domains were simulated by reversing the sign of the piezoelectric tensor while keeping all other parameters fixed, and representative results for $f_{1}=f_{2}=10\;C/\text{nm}$ are presented here. The detailed constitutive formulation, numerical implementation, and material parameters can be found in the Supplementary Materials and table S1.

The interpretation of these simulations is grounded in the established relationship between contact mechanics and friction. Within widely adopted contact-mechanics frameworks, such as the Bowden-Tabor model, the friction force scales approximately with the contact area according to $F_{f}=\tau A_{\text{contact}}$, where τ and $A_{\text{contact}}$ denote the interfacial shear strength and contact area, respectively (*33*–*35*). Under identical tip material, ambient conditions, and sliding parameters, variations in τ are expected to be secondary compared to polarity-dependent changes in contact area. The leading-order contribution to friction contrast arises from the contact-area ratio. The simulated contact-area asymmetry therefore establishes a physically consistent mechanical rationale linking the flexoelectrically modified indentation response to the frictional contrast exploited in PdFM.

Figure 2B plots the simulated indentation depth and contact area as a function of the applied normal force. The results reveal that, under the same normal load, the up-poled domain undergoes deeper indentation and forms a larger contact area compared to the down-poled domain. This difference reflects a modest increase in effective electromechanical stiffness for the down-poled case. The trend is consistent with experimental PdFM measurements in Fig. 1, where friction signals vary asymmetrically with domain orientation. Although the present simulations do not explicitly model sliding friction, as the tangential contact is treated as frictionless, they quantify the polarity-dependent contact mechanics that govern single-asperity interactions at the nanoscale. These findings support the interpretation that the contrast observed in PdFM imaging arises from the interplay between the polarity-dependent piezoelectric response and the polarity-independent flexoelectric contribution during local indentation.

### Electrochemomechanical control of friction in ferroelectrics

**Fig. 3.**
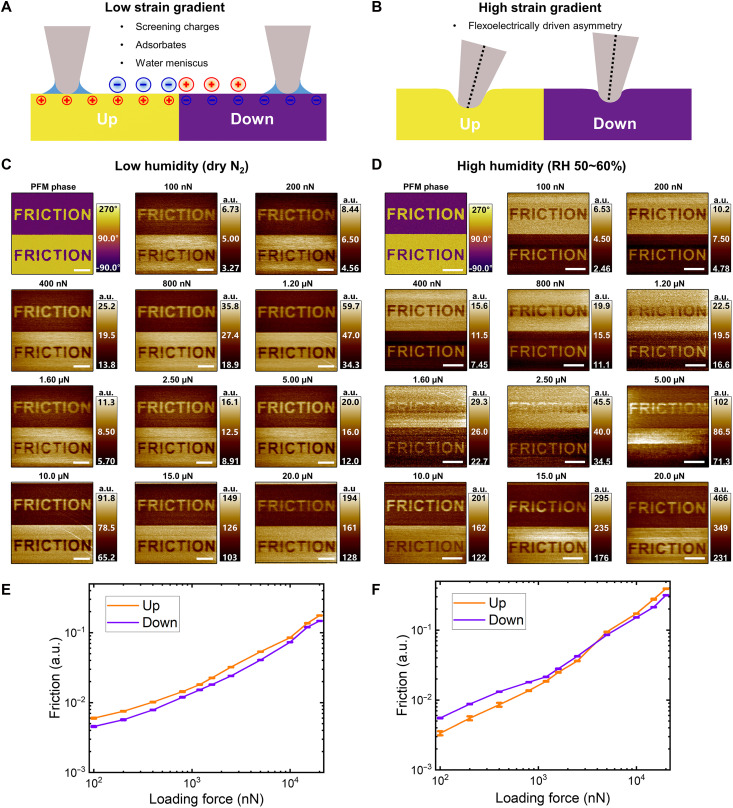
Environment and strain-gradient dependence of PdFM friction contrast. (**A**) At low strain gradient, the flexoelectric contribution is reduced, with screening effects of electrostatic boundary conditions and surface adsorbates predominantly influencing the friction signal. (**B**) Under high strain gradients, the flexoelectric effect dominates, affecting the indentation depth, contact area, and tilt angle of the AFM tip, which differ between up and down domain orientations. (**C** and **D**) Vertical PFM phase images following polarization switching of a PbTiO_3_ thin film and subsequent force-dependent friction images obtained at low and high relative humidity. While the friction contrast remains a stable and thus unambiguous indicator of the polarization orientation at low humidity (dry N_2_ atmosphere), it becomes reversed at high humidity [50% to 60% relative humidity (RH)] in a regime of low strain gradient. (**E** and **F**) Plots of the friction signal versus applied force (logarithmic scale) corresponding to (C) and (D), illustrating the friction trend in each environment. Scale bars, 2 μm.

Since, in PdFM, the lateral signal recorded by the PSPD reflects a convolution of interfacial friction with contributions from near-surface force fields, to better understand the origin and reliability of the friction asymmetry as an imaging marker, we next investigated the same PbTiO_3_ thin film under different relative humidity conditions and varying applied stress, as shown in Fig. 3. At low normal loads (small strain gradients), it is well established that electrostatic boundary conditions and adsorbate-mediated interactions can play a substantial role, complicating direct interpretation of the local mechanical response (*36*, *37*). Specifically, as schematically illustrated in Fig. 3A, polarization-dependent screening charges and environment-dependent adsorption modulate the tip-sample forces; furthermore, in humid air, a water meniscus (a thin layer of water that forms between the tip and the surface) mediates contact, altering contact stiffness and dissipation, and thereby potentially affecting the friction signal. For instance, previous studies have shown that friction signals depending on the polarization configuration in ferroelectric single crystals can vary both in magnitude and sign under the influence of surface conditions (*38*–*40*).

To counteract these undesired effects on the friction signal, it is necessary to enhance the relative contribution of the intrinsic ferroelectric surface polarization—as opposed to screening charges or adsorbate-mediated forces—which can be achieved by increasing the strain gradient. As schematically illustrated in Fig. 3B, higher loads enlarge the true tip-sample contact area and increase the local strain gradient beneath the tip, thereby amplifying the flexoelectric polarization and making it substantial enough to modify the local mechanical response (e.g., contact stiffness and hardness) and dominate over surface electrostatic and adsorbate effects. Under these conditions, the measured friction contrast should thus be governed primarily by the polarization orientation.

For experimental verification, we conducted humidity-dependent friction imaging under environmentally controlled conditions using a flow-mixing controller interfaced directly to the microscope for automated measurements (*41*, *42*). Friction images were acquired while varying the applied strain gradient (by adjusting the loading force) at the same region of the sample after electrical switching of both up and down target domain patterns (Fig. 3, C and D). In the low-humidity scenario (dry N_2_ environment at ~0% RH), we gradually increased the loading force from 100 nN to 20 μN (Fig. 3C) and then decreased it from 20 μN back to 100 nN (see fig. S6). Under these conditions, even at low strain gradients, the role of surface adsorbate effects should be limited. Throughout the measurement cycle, we observe that ferroelectric up domains consistently show higher friction than down domains, making the friction signal a reliable and unambiguous imaging marker of the polarization orientation.

In the higher humidity scenario (Fig. 3D), we maintained the relative humidity between 50 and 60%, across a similar range of loading forces. However, in this measurement cycle, we observe very different friction contrast behavior. In the relatively low loading force regime, up to 2.5 μN, ferroelectric down domains exhibit higher friction than up domains. This trend reverses once loading forces beyond 5 μN are reached, with up domains now exhibiting higher friction signals than down domains. This contrast reversal is clearly related to the force regime since when we decreased the loading force from 20 μN to 100 nN on the same sample region (fig. S7), we once again recovered the initial friction contrast.

The observation that down domains exhibit higher friction signals in the low loading force regime, while up domains show higher friction signals in the high loading force regime, confirms that complex surface screening conditions and electrochemomechanics predominantly influence friction in the low strain gradient regime. Consequently, at elevated humidity levels, establishing a sufficiently high strain gradient is critical for the unambiguous imaging of ferroelectric polarization domains via friction contrast. This requirement is demonstrated in Fig. 3 (E and F), which present friction signals plotted logarithmically against the loading forces for both scenarios. In practical terms, by simply adjusting the applied force, we can selectively access each of these regimes: At low forces, the friction contrast is governed by extrinsic factors (screening charges and adsorbates), whereas at high forces, the friction contrast reflects the intrinsic polarization orientation.

**Fig. 4.**
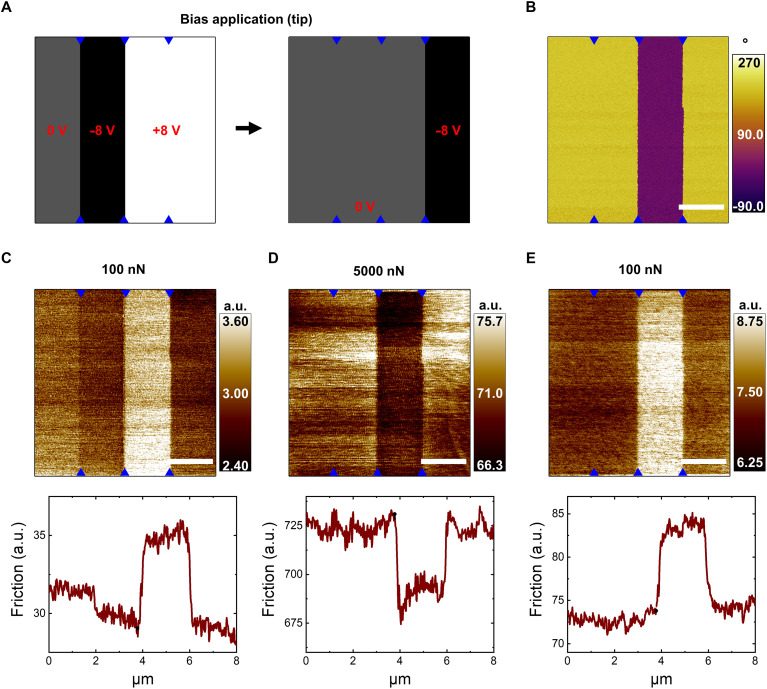
Resettable, load-controlled transition from surface state–dominated to polarization-determined friction on PbTiO_3_. (**A**) Schematic illustration of the bias-writing sequence used to generate four distinct surface states on the PbTiO_3_ thin film by applying sequential voltages to the AFM tip. The four states correspond to the pristine (up domain), overscreened-up state (negative bias applied to the pristine state), down-domain state (positive bias applied to the pristine state), and switched-up state (sequential positive and negative biases applied to the pristine state). (**B**) Corresponding vertical PFM phase image exhibiting clear 180° contrast between oppositely polarized domains. (**C** to **E**) Friction signals and corresponding line profiles acquired under different loading forces on the four engineered surface states. At a low load (100 nN), four distinct contrast levels are observed, corresponding to four different surface states (C). Under a high load (5 μN), only two contrast levels remain, directly mapping to the ferroelectric polarization orientation regardless of the prior surface state (D). When the load is reduced back to 100 nN, the two-level contrast persists, indicating a load-induced reset of near-surface screening and adsorbates to the underlying polarization state (E). Scale bars, 2 μm.

To further investigate the interaction between screening charges and surface adsorbates under controlled strain-gradient conditions, we engineered four distinct surface chemical/electrical states on the PbTiO_3_ sample, as shown in Fig. 4 (A and B): (i) a pristine up-domain state, (ii) an overscreened up-domain state (created by applying a negative bias to the pristine state), (iii) a down-domain state (formed by applying a positive bias to the pristine state), and (iv) a switched up-domain state (generated by sequentially applying a positive and then a negative bias to the pristine state). These engineered surface states exhibit different ratios of adsorbed species and concentrations of screening charges, consistent with previous AFM and x-ray photoelectron spectroscopy studies of ferroelectric surfaces (*21*).

As shown in Fig. 4C, when these four distinct surface states are imaged simultaneously with a low loading force (100 nN), four different friction signal levels are observed, indicating that the surface screening state plays a key role in friction contrast formation. However, when the same surface states are imaged with a higher loading force (5 μN), only two friction signal levels are detected, correlating directly with the polarization state: higher friction in up domains and lower friction in down domains (Fig. 4D). Furthermore, returning to a low loading force (100 nN) after scanning at high loading force still results in only two distinct friction signal levels (Fig. 4E), but inverted. These findings suggest that, in the low loading force regime, electrostatic interactions with screening charges are the dominant contributors to the friction signal and can be modulated by controlling environmental conditions. However, the surface screening states can be “reset” to reflect the underlying polarization alone by effectively removing adsorbates through the application of higher loading force. Thus, during high-force scanning, extrinsic contributions from surface screening and adsorbates are largely suppressed, and the frictional contrast becomes dominated by the polarization-dependent flexoelectric response.

PdFM can consistently operate in a flexoelectricity-dominant friction regime, as long as the applied load is above the threshold necessary to induce sufficient flexoelectric polarization but not so high as to cause flexoelectric switching (*9*, *43*), regardless of surface screening conditions, whether humid or dry. This makes PdFM applicable under a variety of surface conditions. Furthermore, even when electrical bias is applied during PdFM, the intrinsic friction trend is preserved, although the signal levels are modulated by the electrically induced domain responses (fig. S8). In addition, we note that while loading force and scan rate affect the degree of friction asymmetry, they do not change the overall friction asymmetry trend (figs. S9 and S10). We also explicitly verified the stability of the surface topography and ferroelectric polarization states in thin films by comparing images before and after scanning (fig. S11). In other words, the dominance of the flexoelectric contribution to friction contrast is robust over a wide range of scanning conditions, provided that the strain gradient is high enough to avoid extrinsic dominance.

### Broad applicability and high-speed functional imaging

To verify the broad applicability of PdFM, we extended these measurements to a range of ferroelectric systems with different symmetries, morphologies, and thicknesses, such as PbTiO_3_ thin films, LiNbO_3_ single crystals and thin films, Li-doped BiFeO_3_ thin films (*44*), and Ca-doped ErMnO_3_ single crystals (*45*, *46*). In all cases, PdFM consistently revealed polarization-dependent friction contrast, with higher friction on up domains and lower friction on down ones.

**Fig. 5.**
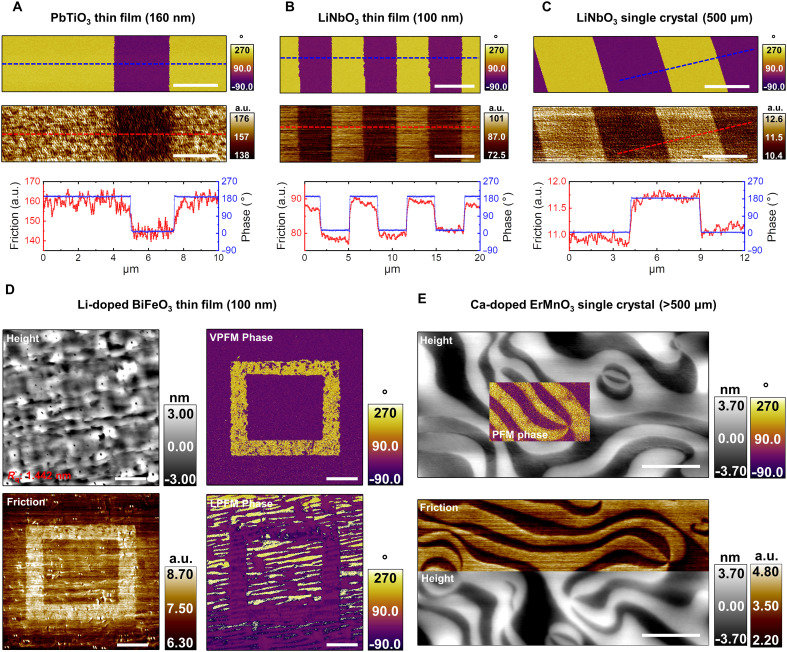
Universality of polarization-derived friction contrast across ferroelectric thin films (PbTiO_3_, LiNbO_3_, Li-doped BiFeO_3_) and single crystals (LiNbO_3_, Ca-doped ErMnO_3_). (**A** to **C**) Vertical PFM phase, PdFM friction image, and corresponding line profiles. PbTiO_3_ thin film (thickness: 160 nm) showing clear friction asymmetry between electrically poled up and down domains (A). LiNbO_3_ thin film exhibiting polarization-dependent friction contrast identical to that observed in PbTiO_3_ (B). Periodically poled LiNbO_3_ single crystal showing one-to-one correspondence between the PFM phase and PdFM friction contrast, confirming that PdFM reliably maps the polarization orientation (C). (**D**) Application of PdFM to a multiferroic Li-doped BiFeO_3_ thin film after double box poling with a biased AFM tip. Despite its island-like surface morphology (roughness: 1.44 nm), the PdFM friction contrast aligns exclusively with the out-of-plane polarization. (**E**) Validation of PdFM on a Ca-doped ErMnO_3_ single crystal, where the friction contrast follows the local out-of-plane polarization component. Scale bars, 2 μm [(A) and (D)] and 4 μm [(B), (C), and (E)].

Figure 5A presents the PdFM images of PbTiO_3_ with a thickness (~160 nm) twice that of the samples used in Figs. 1, 3, and 4 (~80 nm). At increased thickness, we clearly observe friction asymmetry between the up and down domains after electrical poling. PdFM yields consistent polarization-dependent friction contrast in both relatively thin (~80 nm) and thicker (~160 nm) PbTiO_3_ films, demonstrating that the contrast mechanism remains robust across a wide thickness range. These results indicate that PdFM can be universally applied regardless of sample thickness, as long as the strain gradient is optimized to induce friction asymmetry. In the thicker PbTiO_3_ film, for example, a higher effective loading force was used to ensure a sufficient strain gradient, confirming that sample thickness does not fundamentally limit the PdFM contrast when appropriately adjusted. In addition, we successfully applied PdFM to a periodically poled LiNbO_3_ thin film (Fig. 5B) and single crystal (Fig. 5C and fig. S12), both showing higher friction on up-polarized domains, confirming the reproducibility of the PdFM contrast across different ferroelectric systems.

We also applied PdFM to a Li-doped BiFeO_3_ thin film [(Bi_0.99_Li_0.01_)FeO_3_, ~100 nm thick] with an island-like surface morphology (roughness: 1.44 nm). Despite the roughness and the presence of stripe-like ferroelectric domains revealed by vertical and lateral PFM imaging, the PdFM signal exhibited a clear polarization-dependent friction contrast (Fig. 5D and figs. S13 and S14). The friction asymmetry correlates exclusively with the out-of-plane polarization orientation while showing little sensitivity to in-plane domain components or local topography. Moreover, we applied PdFM to hexagonal Ca-doped ErMnO_3_ single crystals [(Er_0.998_Ca_0.002_)MnO_3_, Fig. 5E and fig. S15], which exhibit a domain network (*47*, *48*), with alternating polarization along the depth. Despite the more complex domain geometry, PdFM clearly resolves the near-surface domain configuration through a well-defined polarization-dependent friction contrast. PdFM operates reliably even on surfaces with nanoscale roughness or height steps introduced from the polishing (*45*), demonstrating its tolerance to non-ideal topography. This result demonstrates that PdFM remains highly effective even in morphologically complex and multidomain ferroelectrics, confirming the robustness and selectivity of its polarization-derived friction contrast. Crucially, successful PdFM imaging relies on optimizing the strain gradient—governed by sample thickness and applied pressure—in relation to the dielectric constant (and corresponding flexoelectric coefficient) intrinsic to the material type.

**Fig. 6.**
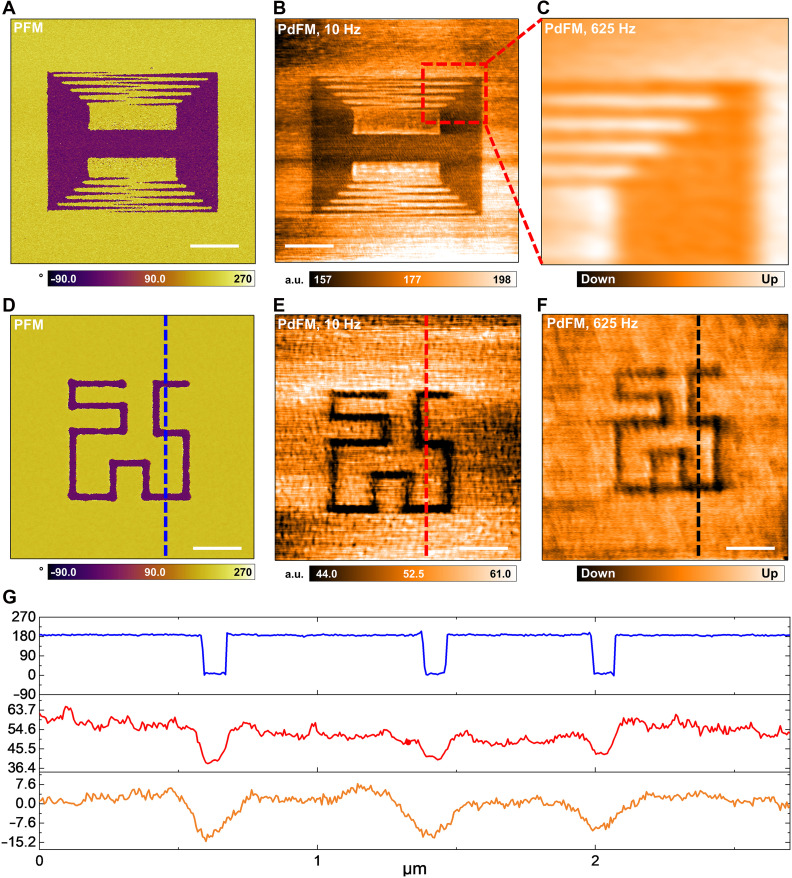
High-speed friction imaging of ferroelectric domains through PdFM. (**A**) PFM phase image of a PbTiO_3_ thin film (80 nm thick) after electrical poling. (**B**) PdFM friction image acquired from the same poled region at a scan rate of 10 Hz, clearly resolving the ferroelectric domain pattern. (**C**) Enlarged PdFM view of the marked area in (B), recorded at 625 Hz, demonstrating that the domain contrast remains discernible even at extreme scanning speeds. (**D**) Vertical PFM phase image obtained from another region of the same sample. (**E**) PdFM friction image of this region acquired at 5 Hz. (**F**) High-speed PdFM image of the same region at 625 Hz. (**G**) Line profiles extracted along the dashed lines indicated in (D) to (F), comparing the PFM phase (blue line) with the PdFM traces acquired at 10 Hz (red line) and 625 Hz (orange line). These profiles confirm consistent polarization-dependent contrast across varying scan speeds, illustrating that PdFM can achieve near-video-rate imaging of ferroelectric domains. Scale bars, 2 μm [(A) and (B)] and 600 nm [(D) to (F)]. The dynamic evolution of the domain imaging at different scan speeds is further visualized in movies S1 to S3.

Last, since frictional imaging does not require additional signal amplification or lock-in averaging, the data acquisition rate of PdFM can be optimized to achieve near-video-rate imaging, representing an enormous increase over conventional PFM imaging speeds. In Fig. 6, we present a proof-of-concept demonstration of such high-speed visualization of ferroelectric domains. After electrical poling, the written domain pattern (verified by PFM; Fig. 6A) was clearly resolved by PdFM at a moderate scan rate (Fig. 6B), and the same features remained well defined even under high-speed scanning [~2.83 frames per second (FPS), 128 × 128 points; Fig. 6C, movie S1]. Additional measurements on another region (Fig. 6, D to F, and movie S2) confirmed that distinct domain contrast is preserved up to the highest scan rate accessible in our setup (625 Hz). Furthermore, PdFM imaging performed on the same region at an even higher frame rate of ~14.2 FPS is shown in movie S3, demonstrating that stable domain contrast can be maintained at video-rate acquisition. As evident from the line profiles in Fig. 6G, increasing the scan speed does not compromise the polarization contrast in PdFM, indicating that the technique offers reliable signal detection even under high-speed conditions. These results establish that PdFM enables high-speed imaging of ferroelectric domains, paving the way toward video-rate data acquisition, polarization dynamics studies, and high-throughput functional imaging in practical applications.

We note previous high-speed PFM studies (*49*–*51*), which have demonstrated the capability of fast imaging of ferroelectric domains through electrically driven piezoresponse. In contrast, PdFM exploits a distinct electromechanical pathway, where local stress gradients induce flexoelectric polarization that gives rise to frictional asymmetry. This voltage-free approach eliminates the need for electrical bias, wiring, or signal amplification, reducing electrostatic artifacts and enabling imaging on samples that are otherwise inaccessible to conventional PFM. PdFM thus extends electromechanical imaging beyond the piezoelectric limit, revealing contrast mechanisms governed by flexoelectricity and interfacial mechanics. Further investigation into these mechanically induced phenomena, such as localized heating or stress-induced phase transitions, will be essential to fully understand and optimize PdFM for diverse applications.

## DISCUSSION

In this work, we introduce PdFM, which uses the friction response as an imaging marker to map out-of-plane ferroelectric polarization orientation under the application of a high strain gradient to the sample. This technique is governed by friction asymmetry resulting from flexoelectrically induced polarization and exhibits universality across various ferroelectric single crystals and thin films. Systematic analysis of the effects of loading force, scan rate, and environmental factors such as humidity reveals the electrochemomechanical control of surface friction in ferroelectrics, defining the operational window for optimal PdFM contrast. PdFM thus provides a simple and high-speed route for voltage-free visualization of ferroelectric domains, achieving scan rates up to 14 FPS and opening pathways for high-throughput mapping of electromechanical functionalities in a broad range of ferroelectrics.

## MATERIALS AND METHODS

### Sample preparation

In this study, the PbTiO_3_ thin film was grown epitaxially using off-axis radio-frequency magnetron sputtering on a TiO_2_-terminated (001)–oriented SrTiO_3_ substrate (CrysTec GmbH). The process began with the deposition of a SrRuO_3_ bottom electrode layer at a temperature of 640°C, using a 100-mTorr O_2_/Ar gas mixture (3:60 ratio), a stoichiometric target, and 80 W of radio frequency power. Subsequently, the PbTiO_3_ film was grown under 180 mTorr of pressure with a 20:29 O_2_/Ar gas ratio and 60-W power at 540°C. To counteract lead volatility, a Pb_1.1_TiO_3_ target with a 10% excess of Pb was used.

A periodically poled lithium niobate (PPLN) single crystal (AR-PPLN test sample, 500 μm thick, from Oxford Instruments Asylum Research) was used as received for PdFM measurements (with alternating ferroelectric domains of opposite polarity). LiNbO_3_ thin films (∼100 nm thick, z-cut) with upward polarization were prepared on Cr/SiO_2_/LiNbO_3_ substrates (NanoLN).

A (Bi_0.99_Li_0.01_)FeO_3_ thin film (~100 nm thick), with an ~80-nm SrRuO_3_ bottom electrode, was epitaxially grown on a SrTiO_3_ (100) substrate using pulsed laser deposition. The deposition was carried out at 650°C under an oxygen pressure of 0.15 mbar, with a KrF excimer laser (λ ~248 nm) at a repetition rate of 5 Hz and a laser fluence of ~0.5 J cm^−2^. After deposition, the film was cooled to room temperature at a rate of 15°C/min under 200 mbar of oxygen.

Single-crystalline (Er_0.998_Ca_0.002_)MnO_3_ was used for the experiments. The crystal was prepared as a half-cylindrical piece, with the curved backside mechanically lapped and the out-of-plane surface carefully lapped and polished. The single crystal was grown using the pressurized floating-zone method (*45*). The sample was then oriented by Laue diffraction and cut to a thickness of about 1 mm with polarization perpendicular to the sample surface, yielding a specimen with out-of-plane polarization. The sample was lapped with an Al_2_O_3_ (9-μm grain size) fluid and polished with a silica suspension (Logitech, SF1 polishing suspension) to achieve a smooth surface. Steps in topography are related to domain-selective etching [see, e.g., (*52*)].

### Scanning probe microscopy

All scanning probe microscopy measurements were performed using a commercial atomic force microscope (Cypher ES/Cypher VRS, Asylum Research). Depending on the sample thickness, robustness, and surface hardness, either metal-coated Si cantilevers (HQ:DPER-XSC11, MikroMasch or 4XC-GG, OPUS) or single-crystalline diamond probes (NM-TC or NC-LC, Adama Innovations) were used for frictional imaging. The loading force was calculated by multiplying the deflection set point (in the position-sensitive photodiode) by the cantilever spring constant and the inverse optical lever sensitivity obtained from force-distance curves. The scan direction was fixed at 90° relative to the cantilever orientation during friction imaging. PFM was performed to image polarization domains at a much lower loading force than that used in PdFM to prevent any sample damage or unintended domain switching during PFM imaging.

### Continuum electromechanical modeling and simulations

Continuum electromechanical simulations were performed using a linear model that includes elastic, dielectric, piezoelectric, and flexoelectric couplings as well as strain-gradient effects. The coupled equations were implemented in Abaqus via a mixed finite-element user subroutine. Axisymmetric indentation simulations were used to evaluate the polarity-dependent electromechanical response under a nanoscale spherical indenter. Full theoretical formulation and material parameters are provided in the Supplementary Materials.

## Supplementary Materials

### The PDF file includes:

Supplementary Text Figs. S1 to S15 Tables S1 and S2 Legends for movies S1 to S3 References
