# Supplementary material for: Nanoscale frictional imaging of ferroelectric domains
Source: Sci Adv. 2026 Jun 26;12(26):eaee0247. doi: 10.1126/sciadv.aee0247 (PMC13308593; doi:10.1126/sciadv.aee0247)
Supplement: Supplementary file 1 — Supplementary Text Figs. S1 to S15 Tables S1 and S2 Legends for movies S1 to S3 References [file sciadv.aee0247_sm.pdf]

Science Advances
AAAS

Supplementary Materials for

Nanoscale frictional imaging of ferroelectric domains

Seongwoo Cho *et al.*

Corresponding author: Seongwoo Cho, seongwoo.cho@unige.ch; Seunghwa Ryu, ryush@kaist.ac.kr; Seungbum Hong, seungbum@kaist.ac.kr; Patrycja Paruch, patrycja.paruch@unige.ch

Sci. Adv. **12**, eaee0247 (2026)
DOI: 10.1126/sciadv.aee0247

The PDF file includes:

Supplementary Text
Figs. S1 to S15
Tables S1 and S2
Legends for movies S1 to S3
References

Other Supplementary Material for this manuscript includes the following:

Movies S1 to S3

Supplementary Text

Continuum modeling of electromechanical coupling

The electromechanical response is described within a linear continuum framework that incorporates elasticity, piezoelectricity, flexoelectricity, dielectricity, and strain-gradient elasticity. The corresponding enthalpy density (*53–55*) is

$$h(\varepsilon, \nabla\varepsilon, E) = \frac{1}{2}C_{ijkl}\varepsilon_{ij}\varepsilon_{kl} + \frac{1}{2}q_{ijklmn}\varepsilon_{ij,k}\varepsilon_{lm,n} - e_{kij}\varepsilon_{ij}E_k - \mu_{lijk}\varepsilon_{ij,k}E_l - \frac{1}{2}\kappa_{ij}E_iE_j \qquad (1)$$

where ε_{ij} denotes the strain tensor, $\varepsilon_{ij,k}$ is the strain gradient, and E_i is the electric field. The coefficients C_{ijkl} , e_{kij} , μ_{lijk} , κ_{ij} and q_{ijklmn} represent the elastic stiffness, piezoelectric, flexoelectric, dielectric permittivity, and strain-gradient elasticity tensors, respectively. In Eq. (1), the first two terms correspond to the elastic contributions associated with strain and strain-gradient effects. The third term describes the piezoelectric response, while the fourth and fifth terms capture the electromechanical couplings arising from flexoelectricity and dielectricity, respectively.

The constitutive relations are obtained by differentiating the enthalpy density with respect to the independent fields. Under isotropic assumptions for elasticity, dielectricity, and flexoelectricity, while retaining the directional dependence of piezoelectricity, the stress σ_{ij}, higher-order stress τ_{ijk}, and electric displacement D_i are expressed as

$$\sigma_{ij} = \frac{\partial h}{\partial \varepsilon_{ij}} = \lambda\varepsilon_{kk}\delta_{ij} + 2\mu\varepsilon_{ij} - e_{kij}E_k \qquad (2a)$$

$$\tau_{ijk} = \frac{\partial h}{\partial \varepsilon_{ij,k}} = l^2(\lambda\varepsilon_{mm,i}\delta_{jk} + 2\mu\varepsilon_{jk,i}) - f_1E_i\delta_{jk} - 2f_2E_j\delta_{ik} \qquad (2b)$$

$$D_i = -\frac{\partial h}{\partial E_i} = \kappa E_i + e_{ijk}\varepsilon_{jk} + f_1\varepsilon_{kk,i} + f_2\varepsilon_{ji,j} \qquad (2c)$$

where λ and μ are the Lamé parameters, l is the strain gradient length scale, κ is the isotropic dielectric permittivity, and f_1 and f_2 are flexoelectric coefficients. The piezoelectric tensor e_{ijk} reduces to three independent nonzero components in crystals with a single polar axis:

$$e_{311} = e_{322} = e_{31}, \qquad e_{333} = e_{33}, \qquad e_{113} = e_{131} = e_{223} = e_{232} = e_{15} \tag{3}$$

with all other components vanishing by symmetry.

The governing equations of the coupled electromechanical problem are derived from the balance of linear momentum and Gauss's law of electrostatics, and are given by

$$\sigma_{jk,j} - \tau_{ijk,ij} + b_k = 0 \tag{4a}$$

$$D_{i,i} - \rho_0 = 0 \tag{4b}$$

where b_k is the body force per unit volume, and ρ_0 is the free charge density. The associated boundary conditions are prescribed on pairwise disjoint subsets of the boundary disjoint parts of the boundary $\partial\Omega$ of the domain Ω. Let n_i denote the outward unit normal.

(1) Traction boundary condition

$$\bar{t}_k = \sigma_{jk}n_j - \tau_{ijk,i}n_j - D_j\left(\tau_{ijk}n_i\right) + (D_l n_l)n_i n_j \tau_{ijk} \quad on\ \partial\Omega_t \tag{5a}$$

(2) High order traction boundary condition

$$\bar{r}_k = \tau_{ijk}n_i n_j \quad on\ \partial\Omega_r \tag{5b}$$

(3) Surface charge boundary condition

$$\bar{\omega} = D_i n_i \quad on\ \partial\Omega_\omega \tag{5c}$$

(4) Displacement boundary condition

$$\bar{u}_i = u_i \quad on\ \partial\Omega_u \tag{5d}$$

(5) Normal derivatives boundary condition

$$\bar{v}_i = Du_i = u_{i,j}n_j \quad on\ \partial\Omega_v \tag{5e}$$

(6) Electric potential boundary condition

$$\bar{\phi} = \phi \quad on\ \partial\Omega_{\phi} \tag{5f}$$

The boundary partitions are mutually exclusive and collectively exhaustive, satisfying

$$\partial\Omega_t \cup \partial\Omega_u = \partial\Omega, \qquad \partial\Omega_r \cup \partial\Omega_v = \partial\Omega, \qquad \partial\Omega_\omega \cup \partial\Omega_\phi = \partial\Omega$$

$$\partial\Omega_t \cap \partial\Omega_u = \emptyset, \qquad \partial\Omega_r \cap \partial\Omega_v = \emptyset, \qquad \partial\Omega_\omega \cap \partial\Omega_\phi = \emptyset$$

Eqs. (5a)–(5c) specify Neumann-type conditions, while Eqs. (5d)–(5f) define Dirichlet-type conditions.

The material parameters used in this study are summarized in Table S1. Given the difficulty of accurately measuring the intrinsic properties of $PbTiO_3$, the adopted values were chosen to be of the same order of magnitude as those commonly reported for ferroelectric perovskites, thereby ensuring physical plausibility while allowing the analysis to emphasize qualitative trends. To examine the role of flexoelectricity, the flexoelectric coefficients were systematically varied over the set $[0,1,5,10,20,50,100] \times 10^{-9}$ C/m, covering regimes where the flexoelectric effect is absent, comparable to the piezoelectric effect, or dominant. The influence of spontaneous polarization was further investigated by performing simulations for both upward- and downward-poled domains, implemented by reversing the sign of the piezoelectric tensor while keeping all other parameters fixed.

Mixed finite element method

To numerically solve the governing partial differential equations described in the previous section, we employ a mixed finite element formulation (*56, 57*). The presence of strain-gradient

terms in the enthalpy density introduces higher–order derivatives of the displacement field. A displacement–potential formulation would therefore require C^1–continuous interpolation, which is not practical in standard finite element frameworks. To circumvent this limitation, we reformulate the problem by introducing the displacement gradient as an independent variable and enforcing the compatibility condition weakly through Lagrange multipliers. This strategy allows all unknown fields to be interpolated with C^0–continuous elements.

The starting point of the formulation is the total potential functional, which can be decomposed into mechanical and electrical contributions as

$$\Pi_{total}(\boldsymbol{u}, \phi) = \Pi_{mech}(\boldsymbol{u}, \phi) - \Pi_{elect}(\boldsymbol{u}, \phi) \tag{6a}$$

$$\Pi_{mech}(\boldsymbol{u}, \phi) = \int_{\Omega} \frac{1}{2}\sigma_{ij}\varepsilon_{ij}\, dV + \frac{1}{2}\tau_{ijk}\varepsilon_{jk,i} dV - \int_{\Omega} b_i u_i\, dV - \int_{\partial\Omega_t} \bar{t}_i u_i dS - \int_{\partial\Omega_r} \bar{r}_i v_i dS \tag{6b}$$

$$\Pi_{elect}(\boldsymbol{u}, \phi) = \int_{\Omega} \frac{1}{2} D_i E_i\, dV - \int_{\Omega} \rho_0 \delta\phi\, dV - \int_{\partial\Omega_\omega} \bar{\omega}\delta\phi dS \tag{6c}$$

Here, $\boldsymbol{u}$ denotes the displacement field and ϕ the electric potential, with the strain defined as $\varepsilon_{ij} = 1/2\left(u_{i,j} + u_{j,i}\right)$ and the electric field as $E_i = -\phi_{,i}$.

To remove the need for C^1 continuity, we introduce an auxiliary field ψ_{ij} representing the displacement gradient. The kinematic constraint $\psi_{ij} = u_{i,j}$ is enforced weakly by bulk and boundary Lagrange multipliers α_{ij} and γ_{ij}. On the boundary, the tangential relation $\psi_{ij}^t = u_{i,j}^t$ is imposed, where the superscript t denotes the tangential component with respect to the outward normal of $\partial\Omega$. The resulting mixed functional is given by

$$\Pi_{total}(\boldsymbol{u}, \phi, \boldsymbol{\psi}, \boldsymbol{\alpha}, \boldsymbol{\gamma}) = \Pi_{mech}(\boldsymbol{u}, \phi, \boldsymbol{\psi}) - \Pi_{elect}(\boldsymbol{u}, \phi, \boldsymbol{\psi}) + \Pi_{LM}(\boldsymbol{u}, \boldsymbol{\psi}, \boldsymbol{\alpha}, \boldsymbol{\gamma}) \tag{7a}$$

$$\Pi_{mech}(\boldsymbol{u},\phi,\boldsymbol{\psi}) = \int_{\Omega} \frac{1}{2}\sigma_{ij}\varepsilon_{ij}\, dV + \frac{1}{2}\tau_{ijk}\psi_{jk,i} dV - \int_{\Omega} b_i u_i\, dV - \int_{\Omega_t} \bar{t}_i u_i dS - \int_{\Omega_r} \bar{r}_i v_i dS \quad (7b)$$

$$\Pi_{elect}(\boldsymbol{u},\phi,\boldsymbol{\psi}) = \int_{\Omega} \frac{1}{2} D_i E_i\, dV - \int_{\Omega} \rho_0 \delta\phi\, dV - \int_{\Omega_\omega} \varpi \delta\phi dS \quad (7c)$$

$$\Pi_{LM}(\boldsymbol{u},\boldsymbol{\psi},\boldsymbol{\alpha},\boldsymbol{\gamma}) = \int_{\Omega} \alpha_{ij}(\psi_{ij} - u_{i,j})\, dV + \int_{\partial\Omega} \gamma_{ij}(\psi_{ij}^t - u_{i,j}^t) dS \quad (7d)$$

In this mixed setting, displacement, electric potential, and the auxiliary gradient field are interpolated with standard C^0 finite elements, while the Lagrange multipliers ensure compatibility between displacement and gradient fields

According to the variational principle $\delta\Pi_{total} = 0$, the stationarity condition can be written as

$$\begin{aligned} &\int_{\Omega} \sigma_{ij}\delta\varepsilon_{ij} + \tau_{\mathrm{ijk}}\delta\psi_{jk,i} dV - \int_{\Omega} b_i \delta u_i\, \mathrm{dV} - \int_{\Omega_t} \bar{t}_i \delta u_i dS - \int_{\Omega_r} \bar{r}_i \delta v_i dS \\ &- \int_{\Omega} D_i \delta E_i\, dV + \int_{\Omega} \rho_0 \delta\phi\, \mathrm{dV} + \int_{\Omega_\omega} \varpi\delta\phi dS \\ &+ \int_{\Omega} \delta\alpha_{ij}(\psi_{ij} - u_{i,j})\, dV + \int_{\Omega} \alpha_{ij}(\delta\psi_{ij} - \delta u_{i,j})\, dV \\ &+ \int_{\partial\Omega} \delta\gamma_{ij}(\psi_{ij}^t - u_{i,j}^t) dS + \int_{\partial\Omega} \gamma_{ij}(\delta\psi_{ij}^t - \delta u_{i,j}^t) dS = 0 \end{aligned} \quad (8)$$

The above form enforces both bulk and boundary kinematic constraints exactly. However, with C^0 interpolation, the tangential boundary condition $\psi_{ij}^t = u_{i,j}^t$ can only be approximated. By relaxing this constraint, Eq. (8) reduces to

$$
\begin{aligned}
\int_{\Omega}\sigma_{ij}\delta\varepsilon_{ij} + \tau_{\mathrm{ijk}}\delta\psi_{jk,i}dV - \int_{\Omega} b_i\delta u_i\,\mathrm{dV} - \int_{\Omega_t}\bar{t}_i\delta u_i dS - \int_{\Omega_r}\bar{r}_i\delta v_i dS \\
- \int_{\Omega} D_i\delta E_i\,dV + \int_{\Omega}\rho_0\delta\phi\,\mathrm{dV} + \int_{\Omega_\omega}\bar{\omega}\delta\phi dS \\
+ \int_{\Omega}\delta\alpha_{ij}\left(\psi_{ij} - u_{i,j}\right)dV + \int_{\Omega}\alpha_{ij}\left(\delta\psi_{ij} - \delta u_{i,j}\right)dV = 0
\end{aligned} \tag{9}
$$

Eq. (9) defines the mixed weak form adopted in the present work. The system is solved using a mixed finite element implementation developed in the commercial software Abaqus, where the formulation is realized through a user-defined element (UEL) subroutine (*58*). This enables direct incorporation of the constitutive relations and higher–order terms associated with piezoelectricity and flexoelectricity within a standard FEM framework.

Axisymmetric FEM modeling of indentation contact

Indentation simulations were carried out using an axisymmetric finite element model to evaluate the electromechanical response under a spherical indenter, as illustrated in Fig. S3. The indenter, modeled as a rigid body with a radius of 7 nm, was displaced vertically by 3 nm along the y-axis. The computational domain measured 40 nm in width and 20 nm in height, dimensions sufficiently large to suppress boundary effects. A maximum indentation depth of 3 nm was applied to ensure consistency with the small-deformation assumption adopted in the constitutive modeling.

The contact between the indenter and the material surface was prescribed as surface-to-surface hard contact in the normal direction, preventing overclosure, and frictionless in the tangential direction. In Abaqus, this condition was implemented using pressure-overclosure = HARD for the normal behavior and by setting the coefficient of friction to zero. The bottom boundary of the domain was fixed in the vertical direction, and symmetry constraints were

enforced along the axis of revolution. The finite element mesh consisted of approximately 30000 axisymmetric elements, with local refinement beneath the indenter to capture contact stresses accurately (Fig. S3B).

To investigate the role of domain polarity, two cases were analyzed: upward- and downward-poled domains. The polarity effect was introduced by reversing the sign of the piezoelectric coefficients in the constitutive model, while all other material parameters were held constant. Representative field distributions of the down-poled domain at $f_1 = f_2 = 10 \times 10^{-9}\ C/m$ are shown in Fig. S4, including radial and axial displacements, electric potential, and strain components. Finally, the indentation force difference between up- and down-poled domains at a depth of 2.5 nm was evaluated for varying flexoelectric coefficients, revealing the polarity-dependent electromechanical stiffness (Fig. S5). When $f_1 = f_2 = 0\ C/m$, the flexoelectric effect is absent, and no force difference arises between the two domains. As the coefficients increase, the force difference grows and reaches a maximum near $f_1 = f_2 = 10 \times 10^{-9}\ C/m$, where the flexoelectric contribution becomes comparable to the piezoelectric effect. Beyond this value, the difference decreases again because the flexoelectric response — acting identically in both up- and down-poled domains — dominates over the piezoelectric contribution, which has opposite signs depending on the domain polarity. This behavior highlights that the observed indentation force difference originates from the competition between polarity-dependent piezoelectricity and polarity-independent flexoelectricity.

We note that most of the perovskites oxide ferroelectrics would tend to fall in or close to the optimal force difference regime, since their flexoelectric constants are of the order of tens of nC/m for lead free $BaTiO_3$ and up to the order of tens of μC/m for the highest lead-based systems such as $Pb(Mg,Nb)O_3$-$PbTiO_3$ with corresponding piezoelectric constants of tens pC/N and up to

thousands pC/N, respectively (*3, 59, 60*) — thus maintaining the crucial balance between the two competing contributions to the friction signal during polarization-derived friction microscopy. Indeed, the generality of PdFM as an imaging method across many different such ferroelectrics is borne out by the experimental observations.

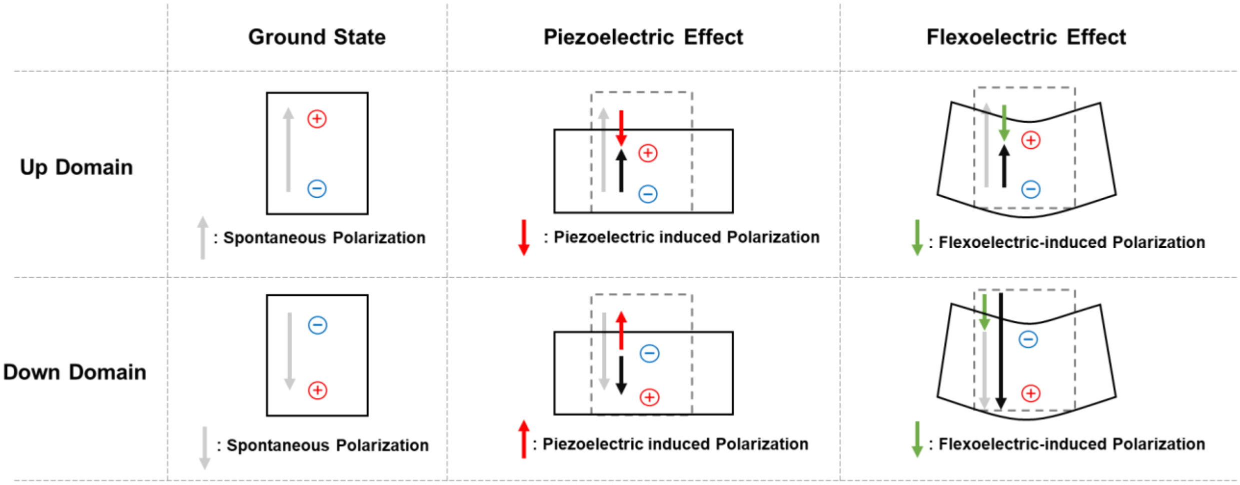

Fig. S1. Schematic of induced piezoelectric effect and flexoelectric effect under mechanical deformation. We note that while the flexoelectric effect is independent of the initial ferroelectric polarization, the piezoelectric effect relies on it.

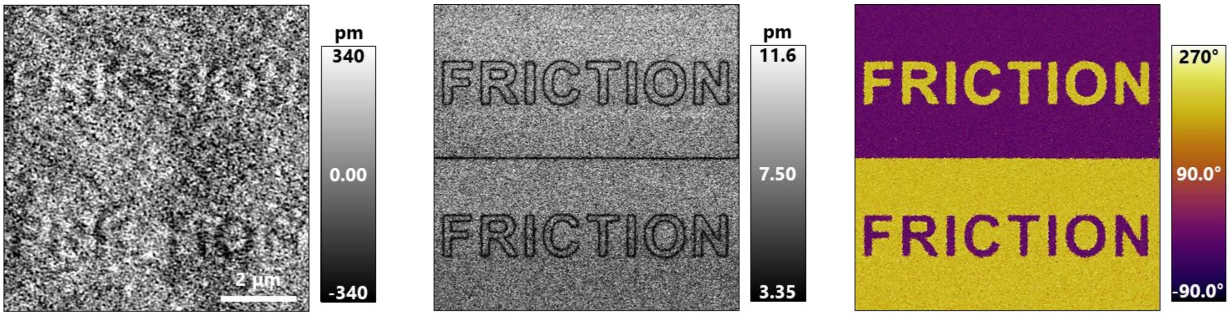

Fig. S2. Height, vertical PFM amplitude and phase of $PbTiO_3$ thin film after artificial patterning of domains via electrical voltage bias to the AFM tip. PFM phase and amplitude show the clear switching from pristine up domain (yellow) to down domain (purple) under application of tip voltage of 8 V.

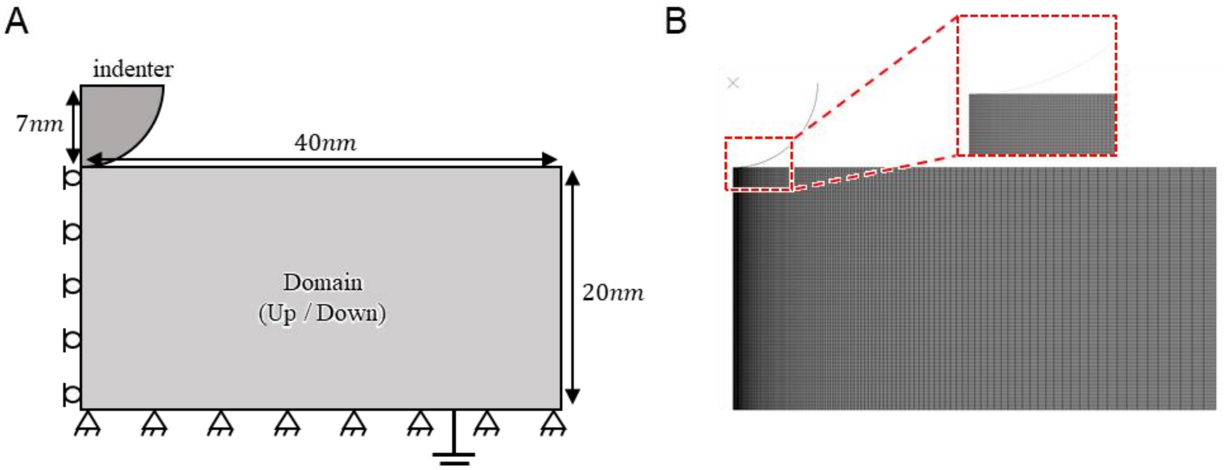

Fig. S3. Axisymmetric finite element model for nanoindentation. (A) Schematic of the computational domain with a spherical indenter of 7 nm radius, model dimensions (40 nm × 20 nm), and boundary conditions. (B) Finite element mesh with local refinement beneath the indenter.

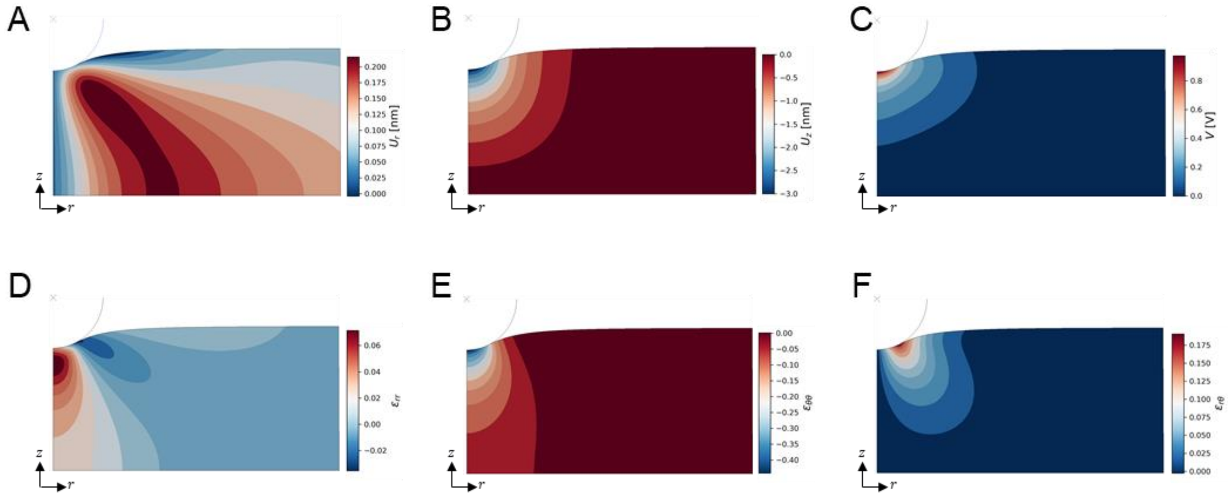

Fig. S4. Finite element results for the down-poled domain at $f_1 = \mathrm{f}_2 = 10\ C/nm$. (A) Radial displacement u_r, (B) axial displacement u_z, (C) electric potential V, (D) radial strain ε_{rr}, (E) hoop strain $\varepsilon_{\theta\theta}$, and (F) shear strain $\varepsilon_{r\theta}$.

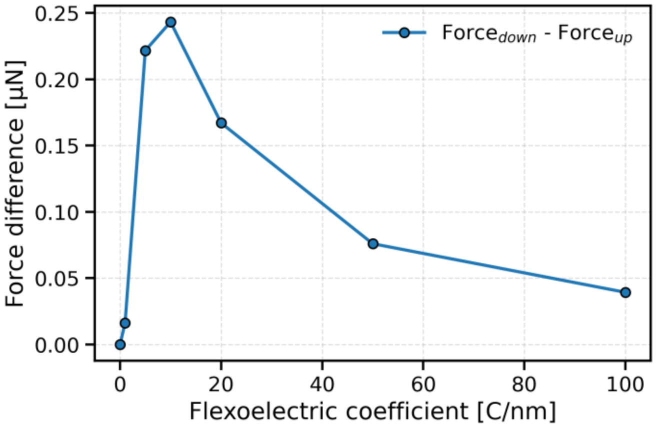

Fig. S5. Indentation force difference between up- and down-poled domains at a depth of 2.5 nm for different flexoelectric coefficients.

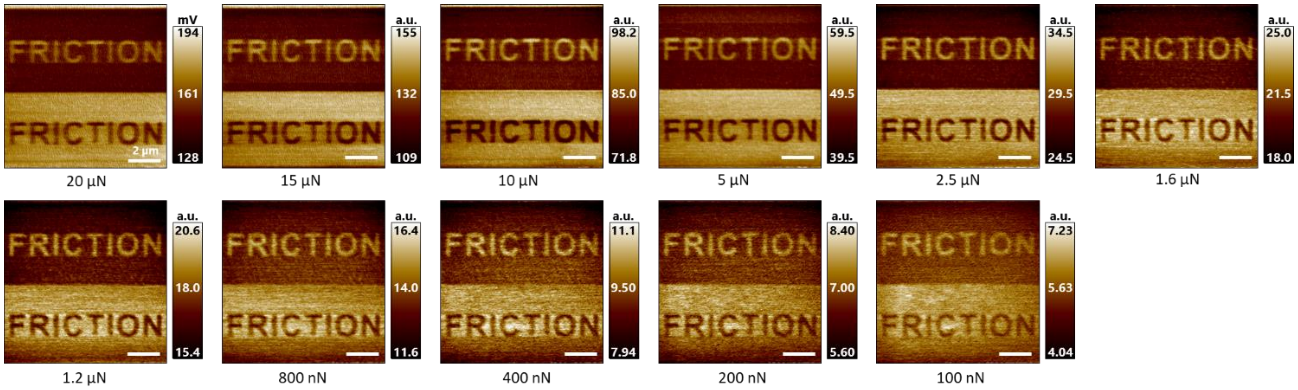

Fig. S6. Friction images obtained while gradually decreasing the loading force under low humidity. These data were acquired after those shown in Fig. 3C. Similar to the case of increasing the load, the frictional trend remains consistent, in contrast to the behavior observed under high humidity.

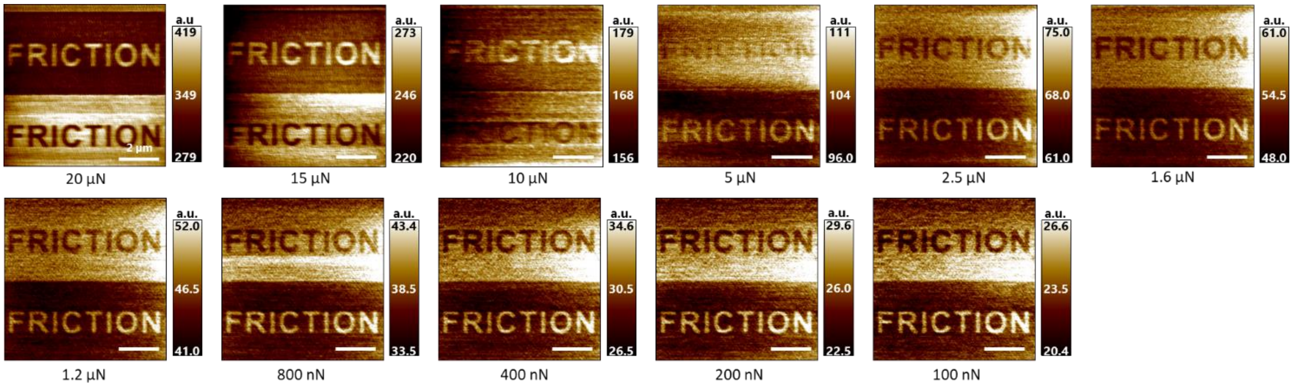

Fig. S7. Friction images obtained while gradually decreasing the loading force under high humidity. These data were acquired after those shown in Fig. 3D. Similar to the case of increasing the load, the frictional trend reverses beyond the threshold load.

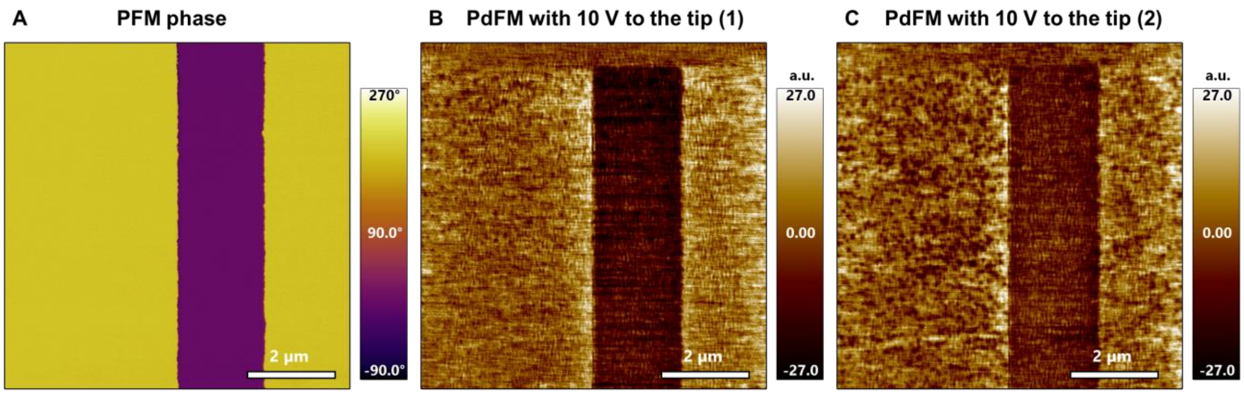

Fig. S8. PdFM under real-time DC bias. (A) PFM phase image acquired simultaneously during PdFM measurements under applied DC bias. (B, C) Sequential PdFM images obtained with a 10 V bias applied to the AFM tip, showing the emergence of dot-like domains induced by local electrical switching.

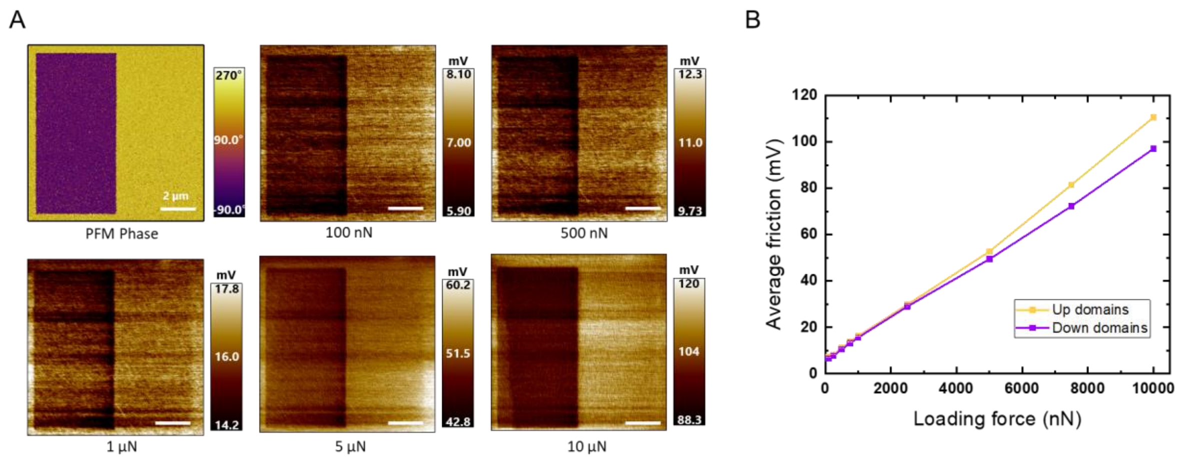

Fig. S9. Frictional behavior on the $PbTiO_3$ ferroelectric surface as a function of increasing loading force. (A) PFM phase image and corresponding friction signals with varying load. (B) Average friction signal as a function of loading force.

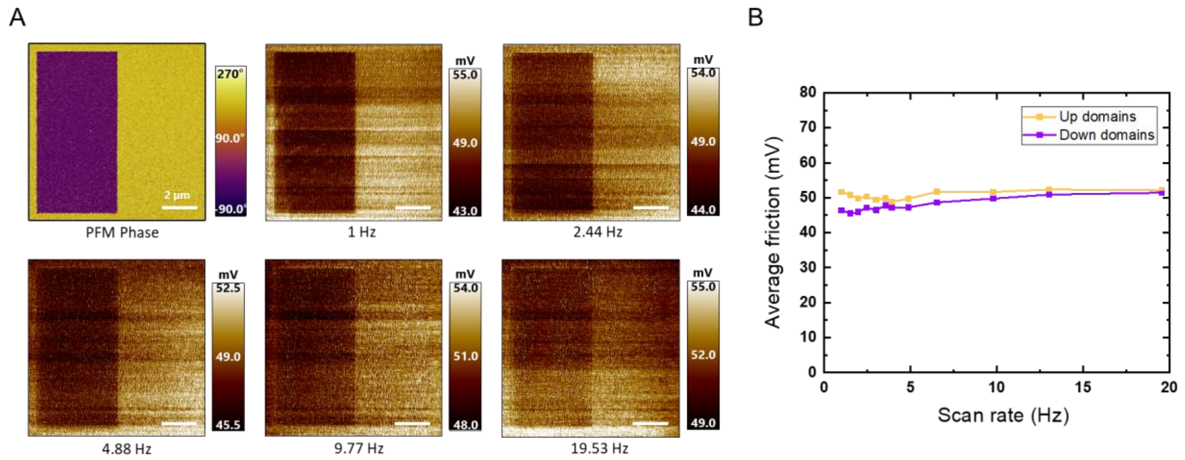

Fig. S10. Frictional behavior on the $PbTiO_3$ ferroelectric surface as a function of increasing scan rate. (A) PFM phase image and corresponding friction signals with varying scan rate. (B) Average friction signal as a function of scan rate.

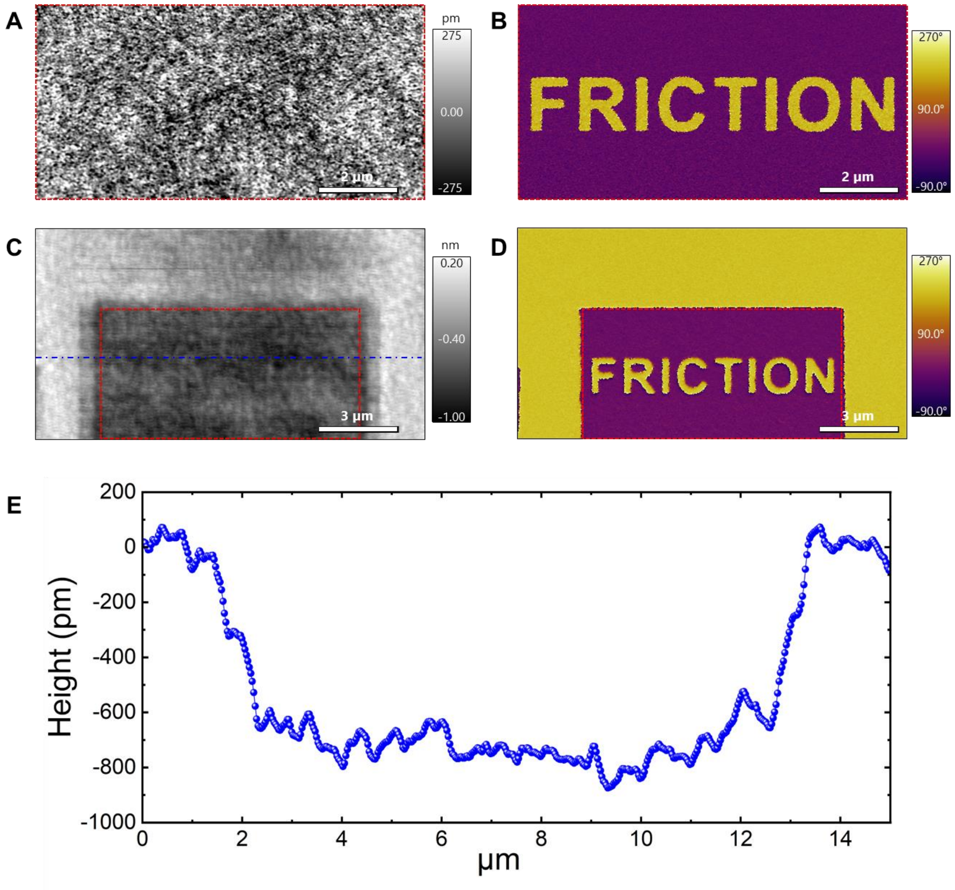

Fig. S11. AFM topography and PFM phase images of a $PbTiO_3$ thin film (~80 nm) before and after PdFM scans. (A, B) Initial topography and PFM phase after electrical switching patterning. (C, D) Topography and PFM phase after 69 consecutive PdFM scans with varying contact forces (100 nN to 20 μN). A pristine background region is included for comparison. Despite a minor surface height reduction (~800 pm) attributed to atomic-scale mechanochemical wear (*61*), the polarization state remains robust, confirming the stability of PdFM under optimized conditions. (E) Cross-sectional height profile extracted from the blue line in (C), averaged over 20 scan lines.

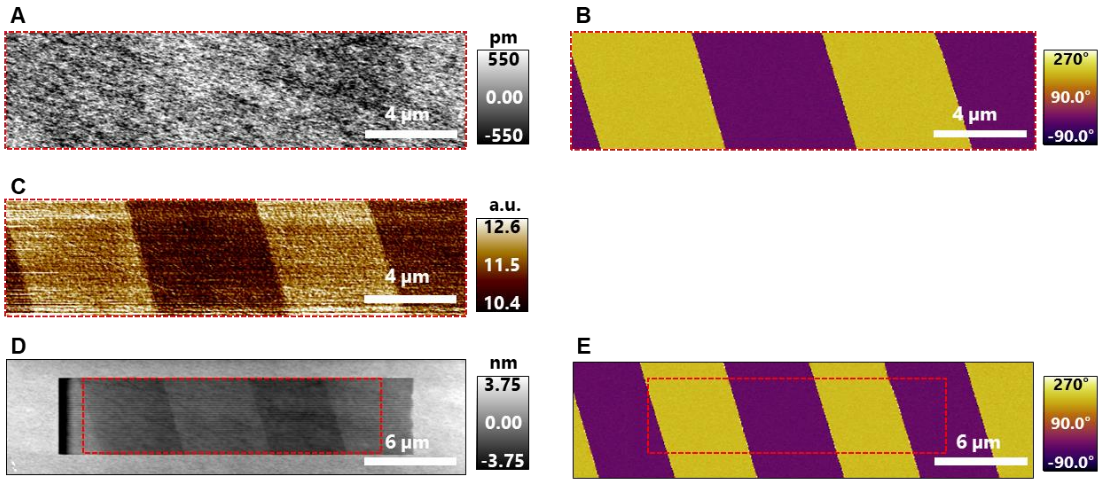

Fig. S12. PdFM imaging of a periodically-poled lithium niobate (PPLN) single crystal. (A, B) Initial topography and PFM phase images acquired before PdFM scanning. (C) PdFM friction image acquired at the same location. (D, E) Topography and PFM phase images acquired after 56 consecutive PdFM scans with contact forces ranging from 500 nN to 5 µN. The area corresponding to the PdFM scans (A–C) is indicated by a red box. A pristine background region is included to facilitate topographic comparison and visualize surface wear.

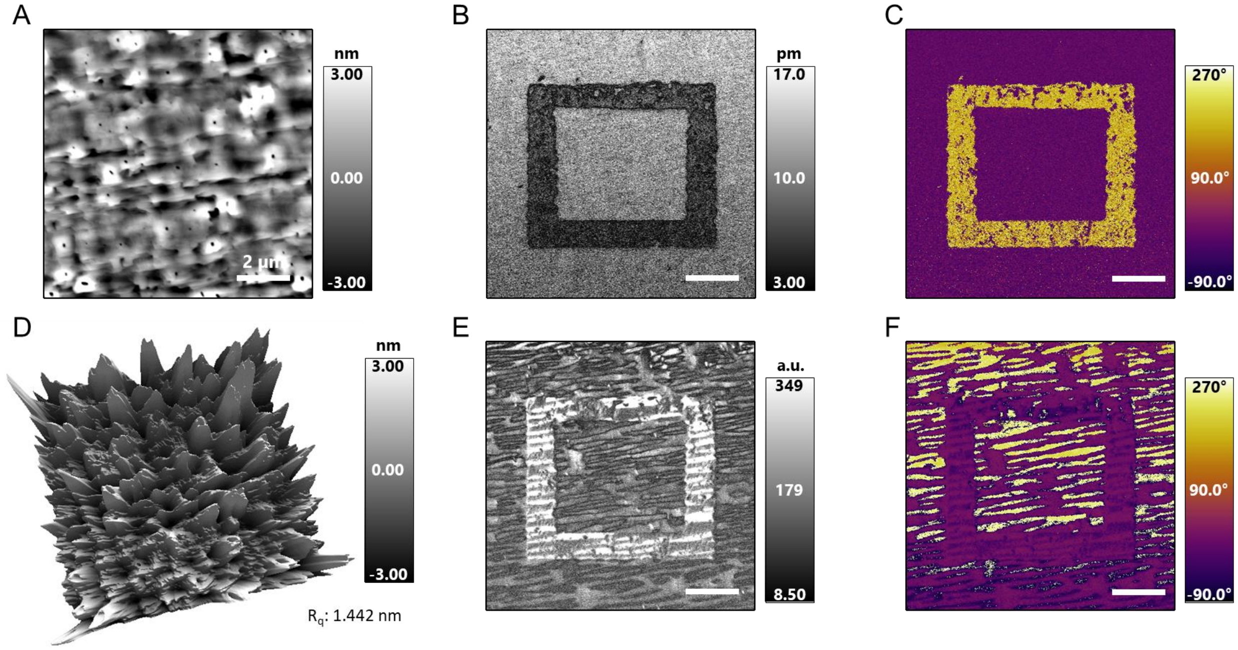

Fig. S13. (A) Height, (B) vertical PFM amplitude, (C) vertical PFM phase, (D) 3D plot of topography, (E) lateral PFM amplitude and (F) lateral PFM phase of a $Bi_{0.99}Li_{0.01}FeO_3$ film after electrical poling. The pristine sample exhibits an out-of-plane down-oriented monodomain configuration and alternating in-plane domains. Note that the images in panels (A), (C), and (F) are identical to those shown in Fig. 5D in the main text.

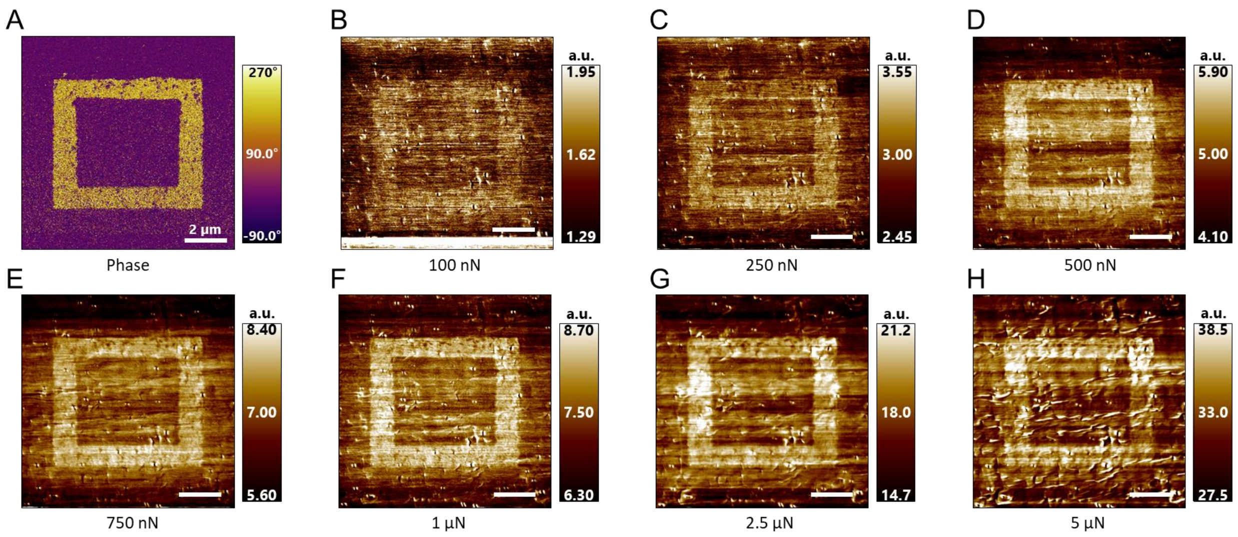

Fig. S14. Polarization-derived friction microscopy of a $Bi_{0.99}Li_{0.01}FeO_3$ film. (A) Vertical PFM phase, PdFM images of (B) 100 nN, (C) 250 nN, (D) 500 nN, (E) 750 nN, (F) 1 µN, (G) 2.5 µN and (H) 5 µN. The PdFM image in panel (F) is identical to the friction image shown in Fig. 5D in the main text. Note that the images in panels (A) and (F) are identical to the vertical PFM phase and friction images shown in Fig. 5D in the main text, respectively.

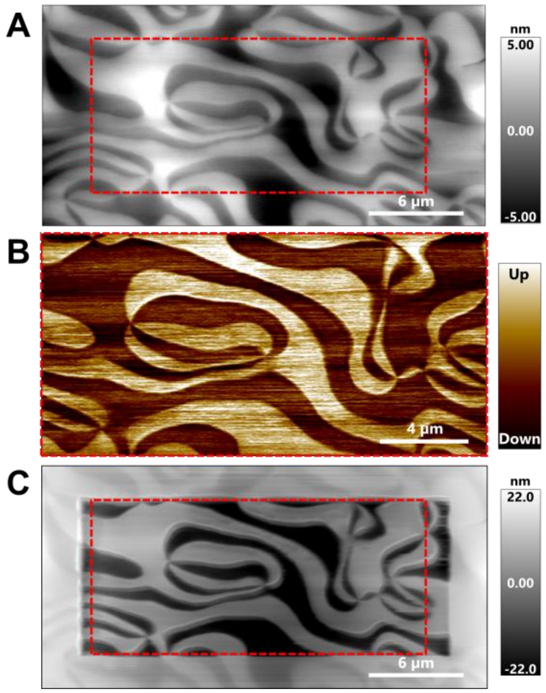

Fig. S15. PdFM imaging of a Ca-doped $ErMnO_3$ single crystal. (A) Topography acquired before the PdFM scan. (B) PdFM friction image visualizing out-of-plane ferroelectric domains. (C) Topography acquired after 50 consecutive PdFM scans with a contact force of 5 µN. The region where the PdFM scan was performed is indicated by a red box.

Table S1. List of material properties

Parameter	Notation	Unit	Value
Lamé constant	λ	GPa	86.4
-	μ	GPa	37.0
Strain gradient length scale	l	nm	2
Dielectric constant	κ	$\mathrm{C/(V \cdot m)}$	0.1
Piezoelectric coefficient	e_{31}	$\mathrm{C/m^2}$	1.644
	e_{33}	$\mathrm{C/m^2}$	2.792
	e_{15}	$\mathrm{C/m^2}$	3.26
Flexoelectric coefficient	f_1	$\mathrm{C/m}$	$[0, 1, 5, 10, 20, 50, 100] \times 10^{-9}$
	f_2	$\mathrm{C/m}$	$[0, 1, 5, 10, 20, 50, 100] \times 10^{-9}$

Table S2. Specifications of AFM probes used for PdFM imaging.

Probe	Description	Spring constant (N/m, nominal)	Tip radius (nm, nominal)
HQ:DPER-XSC11(D)	Pt-coated Si	42	< 20
4XC-GG	Au-coated Si	100	< 30
NM-TC	Single crystal diamond	350	25
NC-LC	Single crystal diamond	100	20

Movie S1. PdFM imaging of an 80 nm $PbTiO_3$ thin film acquired with a metal-coated AFM tip (HQ:DPER-XSC11). Raw scan movie (128 × 128 pixels, 397 Hz line rate, 2.83 FPS) showing nanoscale ferroelectric domain contrast.

Movie S2. PdFM movie of the 80 nm $PbTiO_3$ thin film obtained using a metal-coated AFM tip (4XC-GG, OPUS). Recorded at 256 × 512 pixels, 625 Hz line rate, 1.19 FPS.

Movie S3. High-speed PdFM imaging of the 80 nm $PbTiO_3$ thin film using a metal-coated AFM tip (4XC-GG, OPUS). Fast-scan movie (256 × 32 pixels, 625 Hz line rate, 14.2 FPS) capturing rapid domain-contrast evolution.

REFERENCES

1. D. Damjanovic, Ferroelectric, dielectric and piezoelectric properties of ferroelectric thin films and ceramics. *Rep. Prog. Phys.* **61**, 1267–1324 (1998).

2. H. Jaffe, Piezoelectric ceramics. *J. Am. Ceram. Soc.* **41**, 494–498 (1958).

3. P. Zubko, G. Catalan, A. K. Tagantsev, Flexoelectric effect in solids. *Annu. Rev. Mat. Res.* **43**, 387–421 (2013).

4. D. Lee, A. Yoon, S. Y. Jang, J.-G. Yoon, J.-S. Chung, M. Kim, J. F. Scott, T. W. Noh, Giant flexoelectric effect in ferroelectric epitaxial thin films. *Phys. Rev. Lett.* **107**, 057602 (2011).

5. G. Catalan, A. Lubk, A. H. G. Vlooswijk, E. Snoeck, C. Magen, A. Janssens, G. Rispens, G. Rijnders, D. H. A. Blank, B. Noheda, Flexoelectric rotation of polarization in ferroelectric thin films. *Nat. Mater.* **10**, 963–967 (2011).

6. F. Li, L. Jin, Z. Xu, S. Zhang, Electrostrictive effect in ferroelectrics: An alternative approach to improve piezoelectricity. *Appl. Phys. Rev.* **1**, 011103 (2014).

7. C. Stefani, E. Langenberg, K. Cordero-Edwards, D. G. Schlom, G. Catalan, N. Domingo, Mechanical reading of ferroelectric polarization. *J. Appl. Phys.* **130**, 074103 (2021).

8. S. Cho, I. Gaponenko, K. Cordero-Edwards, J. Barceló-Mercader, I. Arias, D. Kim, C. Lichtensteiger, J. Yeom, L. Musy, H. Kim, S. M. Han, G. Catalan, P. Paruch, S. Hong, Switchable tribology of ferroelectrics. *Nat. Commun.* **15**, 387 (2024).

9. H. Lu, C.-W. Bark, D. E. de los Ojos, J. Alcala, C. B. Eom, G. Catalan, A. Gruverman, Mechanical writing of ferroelectric polarization. *Science* **336**, 59–61 (2012).

10. W. Ming, B. Huang, S. Zheng, Y. Bai, J. Wang, J. Wang, J. Li, Flexoelectric engineering of van der Waals ferroelectric $CuInP_2S_6$. *Sci. Adv.* **8**, eabq1232 (2022).

11. H. Liu, Q. Lai, J. Fu, S. Zhang, Z. Fu, H. Zeng, Reversible flexoelectric domain engineering at the nanoscale in van der Waals ferroelectrics. *Nat. Commun.* **15**, 4556 (2024).

12. S. M. Park, B. Wang, L.-Q. Chen, T. W. Noh, S. M. Yang, D. Lee, Flexoelectric control of physical properties by atomic force microscopy. *Appl. Phys. Rev.* **8**, 041327 (2021).

13. Y. Heo, B.-K. Jang, S. J. Kim, C.-H. Yang, J. Seidel, Nanoscale mechanical softening of morphotropic $BiFeO_3$. *Adv. Mater.* **26**, 7568–7572 (2014).

14. B. Wang, Y. Gu, S. Zhang, L.-Q. Chen, Flexoelectricity in solids: Progress, challenges, and perspectives. *Prog. Mater. Sci.* **106**, 100570 (2019).

15. W. Peng, W. Meng, Y. Kim, J. Yoon, L. Si, K. Zhao, S. Dong, Y. Hou, C. Xi, L. Pi, A. Singh, A. M. Sanchez, R. Beanland, T. W. Noh, Q. Lu, D. Lee, M. Alexe, Ferroelastic writing of crystal directions in oxide thin films. *Nat. Nanotechnol.* **20**, 1199–1204 (2025).

16. S. V. Kalinin, D. A. Bonnell, Imaging mechanism of piezoresponse force microscopy of ferroelectric surfaces. *Phys. Rev. B* **65**, 125408 (2002).

17. A. Gruverman, M. Alexe, D. Meier, Piezoresponse force microscopy and nanoferroic phenomena. *Nat. Commun.* **10**, 1661 (2019).

18. S. V. Kalinin, A. Rar, S. Jesse, A decade of piezoresponse force microscopy: Progress, challenges, and opportunities. *IEEE Trans. Ultrason. Ferroelectr. Freq. Control* **53**, 2226–2252 (2006).

19. E. Soergel, Piezoresponse force microscopy (PFM). *J. Phys. D Appl. Phys.* **44**, 464003 (2011).

20. J. P. Killgore, L. Robins, L. Collins, Electrostatically-blind quantitative piezoresponse force microscopy free of distributed-force artifacts. *Nanoscale Adv.* **4**, 2036–2045 (2022).

21. N. Domingo, I. Gaponenko, K. Cordero-Edwards, N. Stucki, V. Pérez-Dieste, C. Escudero, E. Pach, A. Verdaguer, P. Paruch, Surface charged species and electrochemistry of ferroelectric thin films. *Nanoscale* **11**, 17920–17930 (2019).

22. I. Gaponenko, L. Musy, N. Domingo, N. Stucki, A. Verdaguer, N. Bassiri-Gharb, P. Paruch, Local and correlated studies of humidity-mediated ferroelectric thin film surface charge dynamics. *npj Comput. Mater.* **7**, 163 (2021).

23. R. W. Carpick, M. Salmeron, Scratching the surface: Fundamental investigations of tribology with atomic force microscopy. *Chem. Rev.* **97**, 1163–1194 (1997).

24. P. V. Yudin, A. K. Tagantsev, Fundamentals of flexoelectricity in solids. *Nanotechnology* **24**, 432001 (2013).

25. W. Peng, S. Y. Park, C. J. Roh, J. Mun, H. Ju, J. Kim, E. K. Ko, Z. Liang, S. Hahn, J. Zhang, A. M. Sanchez, D. Walker, S. Hindmarsh, L. Si, Y. J. Jo, Y. Jo, T. H. Kim, C. Kim, L. Wang, M. Kim, J. S. Lee, T. W. Noh, D. Lee, Flexoelectric polarizing and control of a ferromagnetic metal. *Nat. Phys.* **20**, 450–455 (2024).

26. L. Wang, S. Liu, X. Feng, C. Zhang, L. Zhu, J. Zhai, Y. Qin, Z. L. Wang, Flexoelectronics of centrosymmetric semiconductors. *Nat. Nanotechnol.* **15**, 661–667 (2020).

27. J. Narvaez, F. Vasquez-Sancho, G. Catalan, Enhanced flexoelectric-like response in oxide semiconductors. *Nature* **538**, 219–221 (2016).

28. X. Ke, Z. Hong, Q. Ma, X. Wen, Z. Wang, S. Yang, L. Zhang, D. Wang, L. Shu, Q. Deng, S. Shen, X. Ren, Y. Wang, Giant flexoelectric coefficients at critical ferroelectric transition. *Acta Mater.* **245**, 118640 (2023).

29. K. Cordero-Edwards, N. Domingo, A. Abdollahi, J. Sort, G. Catalan, Ferroelectrics as smart mechanical materials. *Adv. Mater.* **29**, 1702210 (2017).

30. W. Melitz, J. Shen, A. C. Kummel, S. Lee, Kelvin probe force microscopy and its application. *Surf. Sci. Rep.* **66**, 1–27 (2011).

31. Q. Zeng, Q. Huang, H. Wang, C. Li, Z. Fan, D. Chen, Y. Cheng, K. Zeng, Breaking the fundamental limitations of nanoscale ferroelectric characterization: Non-contact heterodyne electrostrain force microscopy. *Small Methods* **5**, e2100639 (2021).

32. S. Hong, S. Tong, W. I. Park, Y. Hiranaga, Y. Cho, A. Roelofs, Charge gradient microscopy. *Proc. Natl. Acad. Sci. U.S.A.* **111**, 6566–6569 (2014).

33. B. Gotsmann, M. A. Lantz, Atomistic wear in a single asperity sliding contact. *Phys. Rev. Lett.* **101**, 125501 (2008).

34. M. Lantz, S. O'shea, M. Welland, K. Johnson, Atomic-force-microscope study of contact area and friction on $NbSe_2$. *Phys. Rev. B* **55**, 10776–10785 (1997).

35. M. Enachescu, R. Van den Oetelaar, R. Carpick, D. Ogletree, C. Flipse, M. Salmeron, Observation of proportionality between friction and contact area at the nanometer scale. *Tribol. Lett.* **7**, 73–78 (1999).

36. S. V. Kalinin, E. Karapetian, M. Kachanov, Nanoelectromechanics of piezoresponse force microscopy. *Phys. Rev. B* **70**, 184101 (2004).

37. R. K. Vasudevan, N. Balke, P. Maksymovych, S. Jesse, S. V. Kalinin, Ferroelectric or non-ferroelectric: Why so many materials exhibit "ferroelectricity" on the nanoscale. *Appl. Phys. Rev.* **4**, 021302 (2017).

38. L. Eng, J. Fousek, P. Günter, Ferroelectric domains and domain boundaries observed by scanning force microscopy. *Ferroelectrics* **191**, 211–218 (1997).

39. H. Bluhm, U. D. Schwarz, R. Wiesendanger, Origin of the ferroelectric domain contrast observed in lateral force microscopy. *Phys. Rev. B* **57**, 161–169 (1998).

40. C. J. Long, D. Ebeling, S. D. Solares, R. J. Cannara, Friction imprint effect in mechanically cleaved $BaTiO_3$ (001). *J. Appl. Phys.* **116**, 124107 (2014).

41. I. Gaponenko, L. Gamperle, K. Herberg, S. Muller, P. Paruch, Low-noise humidity controller for imaging water mediated processes in atomic force microscopy. *Rev. Sci. Instrum.* **87**, 063709 (2016).

42. I. Gaponenko, L. Musy, S. Muller, P. Paruch, Open source standalone relative humidity controller for laboratory applications. *Eng. Res. Express* **1**, 025042 (2019).

43. B. Wang, H. Lu, C. W. Bark, C.-B. Eom, A. Gruverman, L.-Q. Chen, Mechanically induced ferroelectric switching in $BaTiO_3$ thin films. *Acta Mater.* **193**, 151–162 (2020).

44. J. H. Lee, I. Fina, X. Marti, Y. H. Kim, D. Hesse, M. Alexe, Spintronic functionality of $BiFeO_3$ domain walls. *Adv. Mater.* **26**, 7078–7082 (2014).

45. Z. Yan, D. Meier, J. Schaab, R. Ramesh, E. Samulon, E. Bourret, Growth of high-quality hexagonal $ErMnO_3$ single crystals by the pressurized floating-zone method. *J. Cryst. Growth* **409**, 75–79 (2015).

46. J. Schaab, A. Cano, M. Lilienblum, Z. Yan, E. Bourret, R. Ramesh, M. Fiebig, D. Meier, Optimization of electronic domain-wall properties by aliovalent cation substitution. *Adv. Electron. Mater.* **2**, 1500195 (2016).

47. T. Jungk, Á. Hoffmann, M. Fiebig, E. Soergel, Electrostatic topology of ferroelectric domains in $YMnO_3$. *Appl. Phys. Lett.* **97**, 012904 (2010).

48. T. Choi, Y. Horibe, H. Yi, Y. J. Choi, W. Wu, S.-W. Cheong, Insulating interlocked ferroelectric and structural antiphase domain walls in multiferroic $YMnO_3$. *Nat. Mater.* **9**, 253–258 (2010).

49. R. Nath, Y.-H. Chu, N. A. Polomoff, R. Ramesh, B. D. Huey, High speed piezoresponse force microscopy: < 1 frame per second nanoscale imaging. *Appl. Phys. Lett.* **93**, 072905 (2008).

50. B. D. Huey, R. Nath Premnath, S. Lee, N. A. Polomoff, High speed SPM applied for direct nanoscale mapping of the influence of defects on ferroelectric switching dynamics. *J. Am. Ceram. Soc.* **95**, 1147–1162 (2012).

51. Y. Liu, B. Yu, Z. Liu, D. Beck, K. Zeng, High-speed piezoresponse force microscopy and machine learning approaches for dynamic domain growth in ferroelectric materials. *ACS Appl. Mater. Interfaces* **12**, 9944–9952 (2020).

52. M. Šafránková, J. Fousek, S. Kižaev, Domains in ferroelectric $YMnO_3$. *Czech. J. Phys. B* **17**, 559–560 (1967).

53. R. D. Mindlin, N. N. Eshel, On first strain-gradient theories in linear elasticity. *Int. J. Solids Struct.* **4**, 109–124 (1968).

54. R. D. Mindlin, Polarization gradient in elastic dielectrics. *Int. J. Solids Struct.* **4**, 637–642 (1968).

55. A. K. Tagantsev, Theory of flexoelectric effect in crystals. *Sov. Phys. JETP* **61**, 1246–1252 (1985).

56. F. Deng, Q. Deng, W. Yu, S. Shen, Mixed finite elements for flexoelectric solids. *J. Appl. Mech.* **84**, 081004 (2017).

57. P. H. Serrao, S. Kozinov, A novel 3D mixed finite element for flexoelectricity in piezoelectric materials. *Int. J. Numer. Methods Eng.* **125**, e7500 (2024).

58. Dassault Systèmes, *Abaqus Analysis User's Guide* (Version 6.14, Dassault Systèmes Simulia Corp., 2014).

59. M. Kołodziej, N. Ojha, M. Budziałowski, K. Załęski, I. Fina, Y. K. Mishra, K. K. Pant, E. Coy, Fundamentals of flexoelectricity, materials and emerging opportunities toward strain-driven nanocatalysts. *Small* **20**, e2406726 (2024).

60. W. Ma, L. E. Cross, Flexoelectric polarization of barium strontium titanate in the paraelectric state. *Appl. Phys. Lett.* **81**, 3440–3442 (2002).

61. L. Chen, J. Wen, P. Zhang, B. Yu, C. Chen, T. Ma, X. Lu, S. H. Kim, L. Qian, Nanomanufacturing of silicon surface with a single atomic layer precision via mechanochemical reactions. *Nat. Commun.* **9**, 1542 (2018).
